# Dynamic Equilibrium between the Fluorescent State of Tryptophan and Its Cation-Electron Ion Pair Governs Triplet State Population

**DOI:** 10.1021/jacs.5c10445

**Published:** 2025-08-21

**Authors:** Rhea Kumar, Sufiyan Khan, Deborin Ghosh, Gabriel Karras, Ian P. Clark, Gregory M. Greetham, Thomas A. A. Oliver, Andrew J. Orr-Ewing, Helen H. Fielding

**Affiliations:** † Department of Chemistry, 4919 University College London, 20 Gordon Street, London WC1H 0AJ, U.K.; ‡ School of Chemistry, 1980 University of Bristol, Cantock’s Close, Bristol BS8 1TS, U.K.; § Central Laser Facility, 547739 STFC Rutherford Appleton Laboratory, Didcot, Oxfordshire OX11 0QX, U.K.

## Abstract

Tryptophan is the most efficient fluorophore of the naturally occurring amino acids and is widely used as a fluorescence probe of protein structure and function. As a result of its importance, there have been numerous studies of the ultrafast photochemical dynamics of tryptophan. Nonetheless, these studies have not identified the pathway to the triplet state, which competes with fluorescence emission. Here, we combine femtosecond-to-microsecond time-resolved transient absorption spectroscopy and time-resolved infrared spectroscopy to explore the photochemical pathway from the UV excitation of tryptophan in aqueous solution to the population of the triplet state and its subsequent relaxation. We observe prompt formation of cations and solvated electrons consistent with autoionization to form a cation-electron ion pair. We find that the cation-electron ion pair subsequently decays with time scales that match the fluorescence lifetime of tryptophan in aqueous solution, indicative of a dynamic equilibrium between the fluorescent state and the cation-electron ion pair. We also find that population of the triplet state occurs on the same time scale as the decay of the cation-electron ion pair and fluorescence, indicating that the triplet state is populated either by recombination of a separated cation and electron after a spin flip or by intersystem crossing from the fluorescent state. Regardless of which mechanism dominates, population of the triplet state of tryptophan is governed by the dynamic equilibrium between the fluorescent state and the cation-electron ion pair.

## Graphical abstract

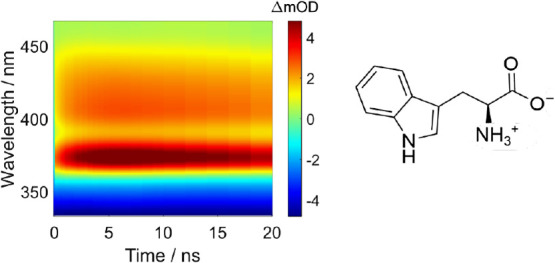

## Introduction

Tryptophan (Trp, Figure 1) is the only essential amino acid that has a fused double-ring side chain. This uniquely large biomolecular motif is responsible for numerous important interactions within proteins that are fundamental to their structure and function.^1^ Trp is the most efficient fluorophore of the naturally occurring amino acids and it is used widely as a fluorescence probe of structural dynamics,^2–6^ solvation,^7,8^ and folding^9^ of proteins. Its fluorescent properties have also been exploited to investigate processes such as energy hopping in microtubules^10^ and to elucidate the mechanism of function in bacteriorhodopsin.^11,12^ Trp also undergoes phosphorescence in solution,^13,14^ and the potential for exploiting Trp phosphorescence to extend the observation window of protein dynamics from the nanosecond time scale of fluorescence to the millisecond-to-second time scales of phosphorescence has been discussed.^14–17^ Nonetheless, the photochemical dynamics of the triplet state that underpin the phosphorescent properties of Trp are still not understood fully.

**Figure 1:**
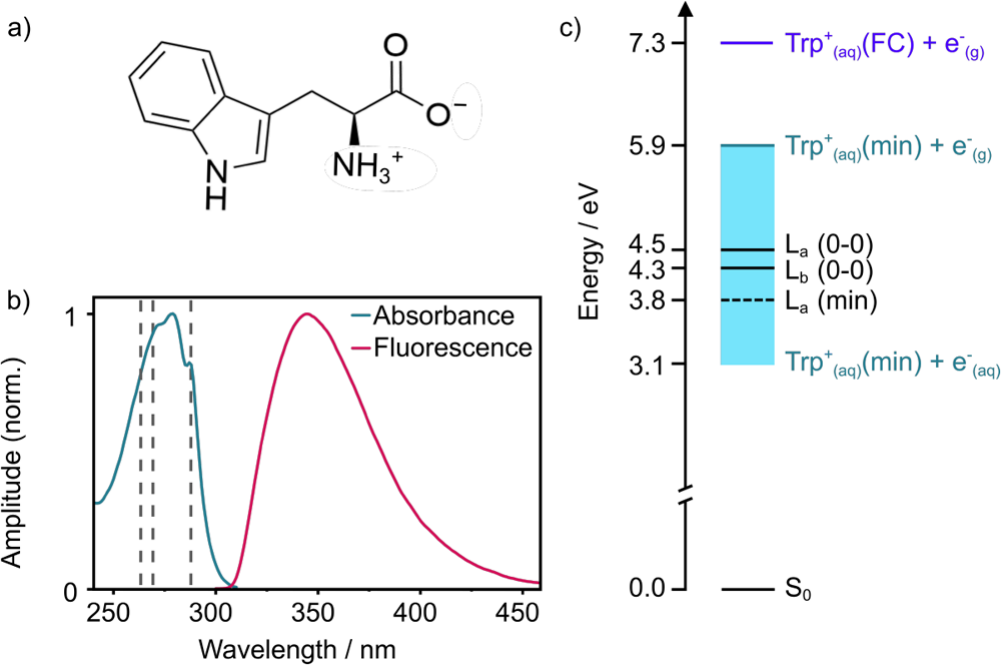
(a) Molecular structure of Trp in its zwitterionic form, as it exists predominantly in unbuffered deionized water. (b) UV–visible absorption and fluorescence spectra of 3.3 mM tryptophan in aqueous solution, normalized to their maxima. The fluorescence spectrum was recorded following photoexcitation at 269 nm. The vertical dashed lines mark the 263 nm, 269 and 289 nm photoexcitation wavelengths employed in this work. (c) Schematic energy level diagram illustrating the ordering of electronically excited states of Trp in aqueous solution. The energy of Trp_(aq)_^+^(FC) + *e*_(g)_^–^ is the vertical ionization energy from S_0_ determined by X-ray photoelectron spectroscopy (PES),^37^ that of Trp_(aq)_^+^(min) + *e*_(g)_^–^ is the adiabatic ionization energy determined by UV PES,^37^ L_a_(0–0) and L_b_(0–0) were estimated from the UV–vis spectrum, L_a_(min) was estimated by subtracting the dynamic Stokes shift^32^ from L_b_(0–0). The shaded area represents the full extent of the solvated electron continuum associated with Trp_(aq)_^+^ in its minimum energy geometry, determined by subtracting the onset of the solvated electron photoelectron spectrum.^38,39^

The UV absorption spectrum of tryptophan comprises transitions to two close-lying and strongly coupled ππ* states, denoted L_a_ and L_b_.^18^ The absorption maxima of these transitions are exquisitely sensitive to the environment of the chromophore and there is ongoing interest in the role of the local environment on the relaxation dynamics following UV photoexcitation of the L_a_ and L_b_ states.^19–21^ Since the chromophore of Trp is the indole moiety, the electronic structure and relaxation dynamics of photoexcited Trp are similar to those of indole.^22,23^ In indole, the static dipole moments of the electronic ground state and the L_b_ state are comparable: 2.09 and 2.13 D, respectively.^24,25^ In contrast, the relatively polar L_a_ state has a dipole moment of 5.4 D.^26^ As a result, the energetic ordering of the L_a_ and L_b_ minima depends on the polarity of the solvent.^27^ In the gas phase and in nonpolar environments, the polar L_a_ state lies higher in energy than the nonpolar L_b_ state; however, polar solvents stabilize L_a_ and induce a dynamic reversal of the energetic ordering of the two states.^28,29^ A similar reordering of energy levels is observed in Trp; it is widely accepted that the L_b_ state of Trp is the adiabatic minimum in the gas phase,^18^ but that in most solutions and proteins the L_a_ state of Trp is the adiabatic minimum and thus the fluorescent state.^29^ The energy level structure and UV absorption and fluorescence spectra of aqueous Trp are presented in Figure 1. Experimental observations of vibronic coupling between the L_a_ and L_b_ states in indole^30,31^ and Trp^32^ support earlier predictions^33^ of a conical intersection (CI) between the two states that facilitates ultrafast (<50 fs) internal conversion in both the gas phase^34^ and in aqueous solution.^35^ A comprehensive study of isolated indole molecules by Giussani et al. found that following initial internal conversion from the photoexcited L_a_ state to the lower-lying L_b_ state through the CI, relaxation back to the ground state could then occur either radiatively (fluorescence) or nonradiatively through a second CI between the L_b_ state and the electronic ground state.^36^ However, the relaxation mechanism is influenced dramatically by the addition of a polar environment. This has been illustrated elegantly by Jaiswal et al., who carried out a combined transient absorption spectroscopy and computational chemistry study of aqueous Trp, in which they observed <50 fs nonadiabatic population transfer from the initially populated L_a_ state to the L_b_ state through an L_a_/L_b_ CI, followed by repopulation of the L_a_ state on time scales of 220 fs and 1.1 ps, attributed to solvent-driven adiabatic stabilization of the L_a_ state.^21^

The well-characterized and pH-dependent fluorescence of Trp has quantum yield Φ ≈ 0.13 in aqueous solution^40^ where the molecule exists in its zwitterionic form, and two lifetimes of around 3 ns (∼80%) and 600 ps (∼20%).^41^ Nonetheless, there is a lack of consensus on the mechanism of relaxation from L_a_ after the solvent-assisted population transfer, with arguments for fluorescence quenching via either intramolecular proton^42–45^ or electron^46,47^ transfer or a coupled process comprising both.^48^ The two lifetime components of aqueous Trp fluorescence are understood to originate from two stable conformations of the alanyl side chain relative to the indole ring, meaning that the donor and acceptor moieties in the charge transfer process experience two distinct separations depending on which conformer the molecule adopts.^41,49,50^ However, the reported addition of a third fluorescence decay component at basic pH (where the amine group is deprotonated) complicates the picture beyond a simple multiconformer model.^51^

An ab initio computational study of gas-phase indole by Sobolewski and Domcke found an additional CI accessible from the ππ* states that allows the system to cross into a dark πσ* state 0.12 eV above L_a_ in the gas phase.^27^ This state was reported to have strong charge transfer character, manifesting as the transfer of electron density from the ring to the NH moiety and resulting in dissociation of the N–H bond. Their results also revealed a further CI between the πσ* state and the ground-state, therefore justifying an efficient nonradiative relaxation mechanism. The same authors later showed that in indole-water clusters, the πσ* state evolves into a charge-transfer-to-solvent state yielding an indole cation and a hydrated electron, which remain connected via a hydrogen bond.^52^ A lifetime of 405 fs has been measured for the relaxation from L_b_ into the πσ* state from gas phase ultrafast spectroscopy of indole by Godfrey et al.^53^ who later found that the relaxation channel involving this state became significant only at wavelengths of 263 nm and shorter,^54^ in agreement with prior studies.^55,56^ However, time-resolved liquid-jet photoelectron spectroscopy measurements carried out by Kumar et al. reported evidence of the πσ* state following photoexcitation at 266 nm for solvated indole, as well as a 0.5 eV stabilization of solvated L_a_ relative to the gas phase, suggesting that the precise threshold for this nonradiative relaxation channel in aqueous solution is currently not well-defined.^22^ Furthermore, a recent computational study of aqueous indole by Chen et al. found that, unlike the back-and-forth transfer of population between the two ππ* states, transitions from the ππ* states into the dark πσ* state occur irreversibly and that a relaxation pathway to the ground-state competes with radiative relaxation.^23^

Several experimental observations of the generation of solvated electrons following photoexcitation to the ππ* manifold confirm that autoionization plays an important role in the relaxation of both photoexcited Trp and indole.^57,58^ Photoionization has been shown to occur via a one-photon process for both indole^59^ and Trp for excitation wavelengths in the range 265–300 nm,^60^ with general agreement that photoelectron ejection occurs on a time scale faster than 200 fs.^22,61^ However, various values for the ionization quantum yield in neutral aqueous solution and at room temperature have been reported, spanning from 0.04 to 0.25.^60,62–64^ Observation of Trp cations with quantum yields comparable to those for solvated electrons corroborates the photoionization process,^65–67^ and supports the suggestion from time-dependent density functional theory and quantum mechanics/molecular mechanics (TDDFT QM/MM) calculations of solvated indole that photoionization competes with other relaxation channels.^68^ Again, the mechanism of the process is debated; there have been suggestions of photoejection directly into a water cage acceptor^69^ as well as reports of a multistep process.^70^ Furthermore, both vibrationally relaxed S_1_,^67^ and nonrelaxed prefluorescent S_1_,^44^ have been proposed as precursors to photoionization. A further point of discussion is the photochemical dynamics after ionization; it has been reported that geminate recombination of cations and electrons in indole is insignificant after 600 ps,^61,71,72^ but scavenging of solvated electrons by Trp cations has been reported to have a rate constant of 7.2 × 10^10^ M^–1^ s^–1^.^67^

As well as fluorescence and photoionization, the triplet state is also involved in the electronic relaxation of Trp. Triplet Trp, ^3^Trp, was first identified by Bent and Hayon as a primary photoinduced intermediate in triplet sensitization and quenching measurements.^46^ These authors also found that deprotonation of the amine eliminated the ^3^Trp contribution, suggesting that its population is dependent on a charge transfer interaction involving the NH_3_^+^ group. A luminescence measurement of aqueous Trp in carefully controlled conditions has reported the intrinsic phosphorescence lifetime of ^3^Trp as 1.2 ms.^14^ Transient absorption spectroscopy (TAS) studies have reported ^3^Trp excited state absorptions (ESAs) that are sensitive to the experimental conditions: 450 nm (following 308 nm excitation in pH 7.0 aqueous solution),^15^ 440–445 nm (following 265 nm excitation in pH 5.4 unbuffered aqueous solution),^46^ 430 nm (following 292 nm excitation in pH 7.4 phosphate buffer)^73^ and 420 nm (following 266 nm excitation in pH 5.9 unbuffered aqueous solution).^67^ Although it is agreed that ^3^Trp is formed, the mechanism for its population is still a source of debate. Tsentalovich et al. have suggested that ^3^Trp^+^ is formed via intersystem crossing after intramolecular proton transfer from the NH_3_^+^ group to the indole ring.^15,44^ A recent, detailed TAS and fluorescence lifetime study of the electronic relaxation dynamics of aqueous indole following photoexcitation at 292, 266, and 200 nm, found that beyond that occurring within the 5 ns instrument function, triplet population occurred 2 orders of magnitude more slowly than fluorescence (∼4.5 ns) on a time scale of 110 ns.^72^ It was proposed that the triplet was formed by recombination of fully separated cation-electron ion pairs. Nonetheless, although there have been numerous experimental studies probing the electronic relaxation dynamics of Trp, from early photoionization measurements,^44,63,74,75^ to time-resolved spectroscopy measurements that include TAS,^19,21,45,76^ fluorescence spectroscopy,^77–79^ and photoelectron spectroscopy,^80,81^ the mechanism for the population of ^3^Trp is still unknown. Motivated by this lack of understanding, and its relevance in fluorescence and phosphorescence probing of protein dynamics, we have undertaken a combined femtosecond-microsecond TAS and time-resolved infrared (TRIR) spectroscopy study of aqueous Trp following photoexcitation at 263 nm, 269 and 289 nm.

## Results and Discussion

### Transient Absorption Spectroscopy

TA spectra recorded following 269 nm photoexcitation over the range 0–3 μs using the LIFEtime facility at the Rutherford Appleton Laboratory (RAL) are presented in Figure 2. In the 380–470 nm range, an absorption feature is observed around 430 nm. This feature appears within the first 2 ps and is observed to increase in intensity and change shape during the first 10 ns, before subsequently decreasing in intensity. In the 500–860 nm range, the TA spectra at long times (>3 ns) are dominated by a broad band centered around 700 nm that is characteristic of the absorption spectrum of the solvated electron (Figure S1).^82^ The spectral evolution of this feature at earlier times (<10 ps, Figure S1) does not match the expected transient absorption signatures associated with ballistic ejection of an electron from the parent molecule.^83,84^ The dynamical shifts are slower and more modest, and could be attributed to the formation of a cation-electron ion-pair^72^ or slow ejection of an electron.^85,86^ We do not discuss this mechanistic question further, but hereafter simply refer to the formation of cations and solvated electrons. The presence of solvated electrons is supported by observing this broad band decrease in intensity more rapidly on addition of HCl as a solvated electron quencher (Figure S1). L_a_ excited state absorptions (ESAs) and cation absorptions have also been identified in this spectral range in previous TA spectroscopy studies,^21,45^ and are assumed to contribute to the structure superimposed on the solvated electron absorption band. The broad feature resulting from overlap of the solvated electron band with these other spectral contributions appears to shift toward shorter wavelengths between 2 and 100 ps, before shifting back toward longer wavelengths after around 3 ns.

**Figure 2:**
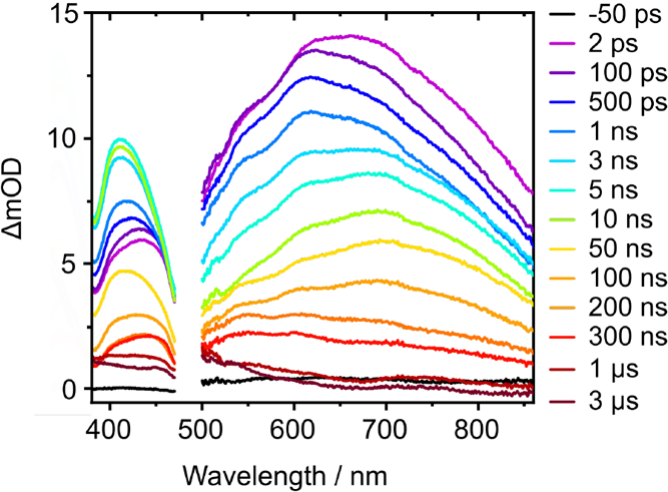
Transient absorption spectra of 3.3 mM aqueous tryptophan following photoexcitation at 269 nm at specified pump–probe delays from 0 to 3 μs. The gap between 470 and 500 nm is the region between white-light continuum (WLC) probe ranges where the probe light intensity is insufficient for good quality measurements.

To improve on the temporal resolution at shorter times and to fill the gap between 470 and 500 nm, we also recorded TA spectra over the range 0–7 ns using the femtosecond laser facility at University College London (UCL). TA spectra following photoexcitation at 269 nm are presented in Figure 3, plotted over four distinct time scales: 0–8 ps, 8–200 ps, 200–600 ps, and 0.6–7 ns. Similar TA spectra following photoexcitation at 289 and 263 nm are presented in the SI (Figures S2 and S3, respectively).

**Figure 3:**
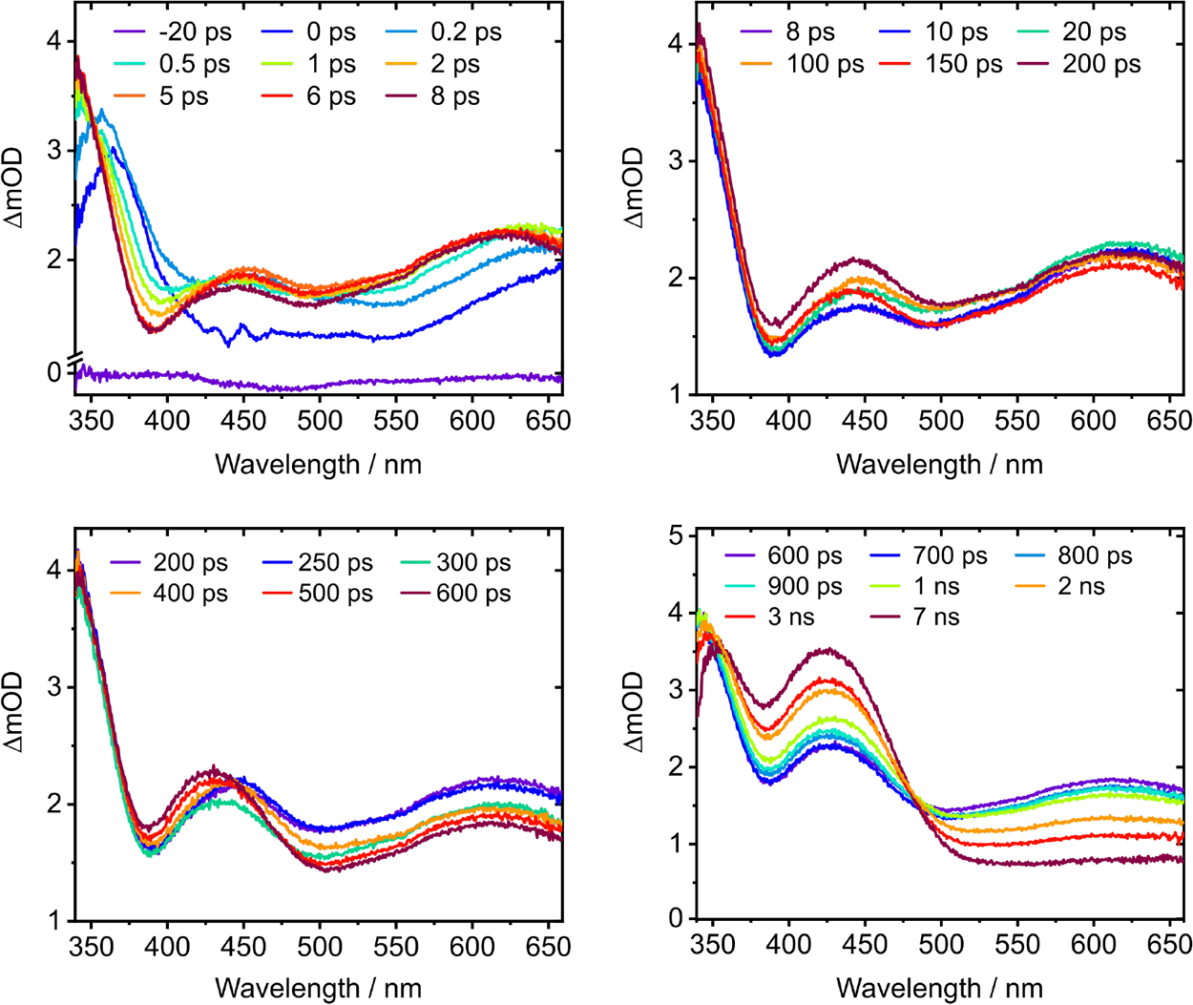
Transient absorption spectra of 3.3 mM aqueous tryptophan following photoexcitation at 269 nm at specified pump–probe delays, plotted over four ranges: 0–8 ps, 8–200 ps, 200–600 ps, and 0.6–7 ns.

In the first 200 fs, a strong ESA is observed centered around 365 nm, as well as a weaker 400–500 nm ESA, together with the broad absorption centered at wavelengths >640 nm characteristic of solvated electrons. During the next 2 ps, the 365 nm ESA appears to shift toward shorter wavelengths until an ESA extending to wavelengths below 340 nm dominates the short wavelength region. Concomitantly, the 400–500 nm ESA evolves into a well-defined ESA centered around 450 nm. The broad absorption band that initially appears to be centered at wavelengths longer than 650 nm broadens and shifts toward shorter wavelengths. This could be consistent with solvation following ionization^87^ or changes in the relative contributions of solvated electron and cation absorptions, and L_b_ and L_a_ ESAs that are known to extend across this region.^21^ By around 8 ps, the ESA extending below 340 nm has appeared to stop shifting, and the 450 nm ESA has begun to shift toward shorter wavelengths. The solvated electron band begins to decrease in intensity, and there is a peak around 620 nm that is consistent with the structure observed across the solvated electron absorption band in the TA spectra presented in Figure 2.

Transient absorption features following UV excitation of Trp in aqueous solution have been discussed thoroughly in the literature and allow us to attribute the 365 nm ESA to the L_b_ state, and the ESAs centered around 450 nm and below 340 nm to the fluorescent L_a_ state.^21^ The concomitant decay of the L_b_ ESA and rise of the L_a_ ESAs, together with an isosbestic point around 354 nm, are consistent with the L_b_ →L_a_ solvent-driven population transfer reported by Jaiswal et al.^21^ A shoulder around 580 nm that becomes more evident after around 1 ps has been identified in an earlier TA study as a Trp^+^ (cation) absorption.^45^ This Trp^+^ absorption can be seen clearly in the 1–3 μs TA spectra recorded with HCl added to quench the solvated electron (Figure S1) which leads us to suspect that the peak observed around 620 nm in Figures 2 and 3 may arise as a result of overlapping Trp^+^ and solvated electron absorption bands. Our 1–3 μs TA spectra of Trp in aqueous solution with HCl also allow us to identify another Trp^+^ absorption around 350 nm. Autoionization to form the cation and a solvated electron following photoexcitation at wavelengths 289–263 nm is consistent with the bottom of the solvated electron continuum associated with Trp^+^ in its minimum energy configuration lying at 3.1 eV (395 nm), determined by subtracting the adiabatic detachment energy (ADE) of the solvated electron from the adiabatic ionization energy of Trp (5.9 eV),^37^ obtained from liquid-microjet photoelectron spectroscopy measurements (Figure 1). The ADE of the solvated electron was estimated from accurate photoelectron spectroscopy measurements.^38,39^

Between 8 and 200 ps, the TA signal that extends below 340 nm barely evolves, but the relative amplitudes of the various overlapping absorption features at longer wavelengths do, and the ESA around 450 nm appears to shift to shorter wavelengths and evolve into an ESA centered around 430 nm. Between 200 and 600 ps, the TA signal between around 500 and 650 nm that comprises contributions from the broad L_a_ ESA, and solvated electron and Trp^+^ absorption bands, decreases in intensity. The TA feature in the 400–500 nm region continues to shift to shorter wavelengths, suggesting that the 430 nm ESA is rising as the 450 nm L_a_ ESA feature decays. There is also a rise in the TA feature that extends below 340 nm. From 600 ps to 7 ns, the Trp^+^ ESA and solvated electron absorption bands decrease in intensity as the 430 nm ESA and the ESA that extends below 340 nm increase in intensity. Together with the isosbestic point around 480 nm, this suggests that the decay of L_a_, the cation and solvated electron are all involved in the population of the photoproduct that gives rise to the 430 nm ESA and the ESA that extends below 340 nm.

The triplet state, ^3^Trp, has been identified as having an ESA around 430 nm in aqueous solution.^15,46,67,73^ However, Haacke and co-workers assigned a 430 nm ESA in their femtosecond TA spectroscopy study of Trp as a zwitterion with the indole moiety protonated by excited state proton transfer from the side chain amine group.^45^ Therefore, to determine whether the absorption band around 430 nm is a ^3^Trp ESA, we recorded additional TA spectra of aqueous Trp over the range 0–8.3 μs following photoexcitation at 290 nm using the LIFEtime facility at RAL, and compared these with equivalent spectra with 0.2 M MnSO_4_ added to quench the triplet (Figure 4). In both sets of TA spectra, the feature centered around 430 nm increases in intensity until around 5 ns before decreasing to just less than half its maximum absorbance by around 500 ns. Between 500 ns and 8.3 μs, the absorbance decreases more rapidly in the spectra recorded with 0.2 M MnSO_4_ added, suggesting that the feature around 430 nm is a ^3^Trp ESA.

**Figure 4:**
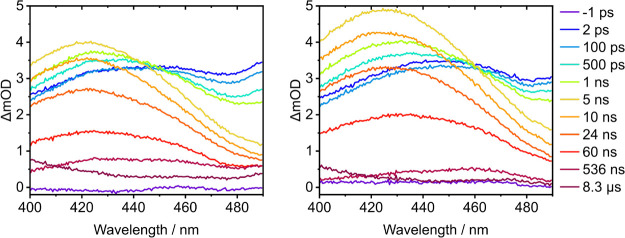
Transient absorption spectra of 3.3 mM tryptophan at specified pump–probe delays from 0 to 8.3 μs following 290 nm photoexcitation in aqueous solution (left) and with the addition of 0.2 M MnSO_4_ (right).

To quantify the dynamics, the UCL TA spectra (Figures 3, S2, and S3) were decomposed into spectral bands associated with the L_a_ and L_b_ ESAs, and solvated electron and Trp^+^ absorption bands, using Gaussian fitting functions in the KOALA software.^88^ It should be noted that the band labeled as the solvated electron absorption band will include a contribution from the broad L_a_ ESA that is known to extend across the whole of our probe region.^21^ First, to capture the dynamics of the L_b_ →L_a_ population transfer at early times (0–15 ps), the 269 nm TA spectra were decomposed into four spectral bands over the range 340–420 nm. The central wavelengths and widths of bands associated with solvated electron and Trp^+^ absorptions, and L_a_ and L_b_ ESAs, are presented in Table 1. Snapshots of the fits are presented in Figure S4, and the kinetic traces obtained by integrating the areas of the spectral bands in the 269 nm TA spectra are presented in Figure 5.

**Table 1:** Central Wavelengths in nm (Full Width at Half-Maxima in Parentheses) of the Gaussian Spectral Bands Fit to TA Spectra of 3.3 mM Tryptophan across the Range 340–440 nm, Following Photoexcitation in Aqueous Solution

*e*_(aq)_^–^	L_a_	L_b_	cation
719 (320)	332 (52)	373 (43)	354 (25)

**Figure 5:**
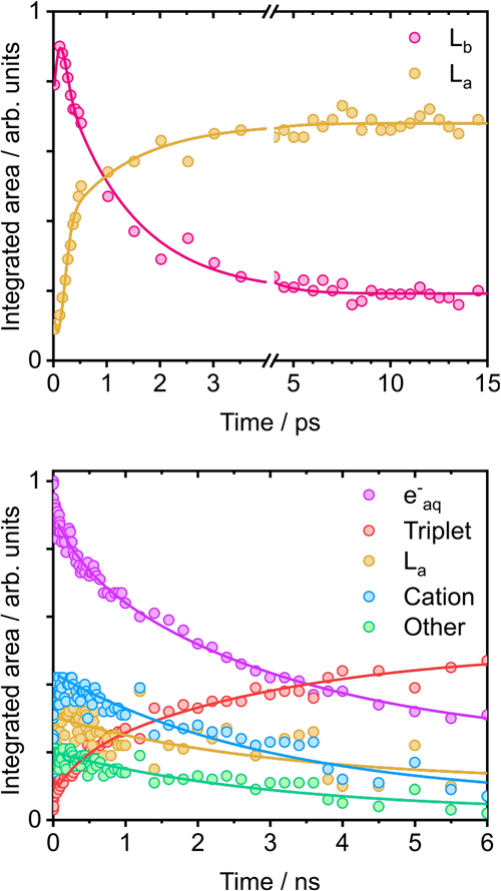
Kinetic traces of the features in the 269 nm TA spectra of aqueous Trp. Over the 0–15 ps range (top), the features of the TA spectra are fit with the parameters listed in Table 1. The integrated areas of the L_b_ and L_a_ spectral bands (circles) have been globally fit to a biexponential convoluted with a Gaussian instrument response function with standard deviation of 200 fs. Over the 0–7 ns range (bottom), the features of the TA spectra are fit with the parameters listed in Table 2. The time-dependent integrated areas of the *e*_(aq)_^–^ and ^3^Trp spectral bands are globally fit to a biexponential function. The integrated areas of the remaining three spectral bands are fitted to biexponential functions constrained to have the same time constants but amplitudes that were allowed to vary.

Fitting a single exponential decay for *t* > 0.3 ps gave a time constant of 1.1 ± 0.1 ps (Figure S5), which is in agreement with the longer of the two time scales reported by Jaiswal et al. for solvent-driven L_b_ →L_a_ population transfer following 284 nm photoexcitation of 36 mM Trp in a phosphate buffer solution at pH 7.4 (220 fs and 1.1 ps).^21^ The temporal resolution of the TA experiments reported by Jaiswal et al. was <30 fs, from which a <50 fs nonadiabatic L_a_ →L_b_ transfer through a conical intersection was determined to precede the solvent-driven L_b_ →L_a_ transfer. Fitting a biexponential decay convoluted with an instrument response function (IRF) with 200 fs standard deviation to our data (Figure 5) gave time constants of <100 fs (IRF limited) and 1.3 ± 0.1 ps. These time scales are in agreement with those reported for nonadiabatic transfer through the L_a_/L_b_ conical intersection and subsequent solvent driven L_b_ →L_a_ population transfer. In our kinetic traces, the rise of the solvated electron signal appears to be faster than the formation of the L_a_ state (Figure S5), which could indicate that autoionization occurs preferentially from the L_b_ state.

To capture the dynamics of triplet population, the 0–7 ns TA spectra were decomposed into five spectral bands over the range 430–660 nm. The central wavelengths and widths of these bands are presented in Table 2 and have been assigned labels according to known solvated electron and Trp^+^ absorptions, and ^3^Trp and L_a_ ESAs. As noted above, the band labeled as the solvated electron absorption band will include a contribution from the broad L_a_ ESA (Supporting Information, Section S4). The absorption centered at 510 nm was included to improve the fit and is most likely required to account for an additional feature of the L_a_ ESA. It is worth highlighting that the same parameters can be used to fit the TA spectra of Trp in aqueous solution excited at all wavelengths reported in this work (289 nm, 269 and 263 nm), and also for Trp in aqueous solution with 0.2 M HCl, 0.5 M KNO_3_, and 0.2 M MnSO_4_, with the exception of the triplet ESA band which is shifted 15 nm to shorter wavelengths in TA spectra recorded with 0.2 M HCl, consistent with previous observations.^44^ Snapshots of the fits are presented in Figures S6–S8, and kinetic traces obtained by integrating the areas of the spectral bands for the 269 nm TA spectra of aqueous Trp are presented in Figure 5.

**Table 2:** Central Wavelengths in nm (Full Widths at Half-Maxima in Parentheses) of the Gaussian Spectral Bands Fit to Transient Absorption Spectra of 3.3 mM Tryptophan across the Range 430–660 nm, Following Photoexcitation in Aqueous Solution, and in Aqueous Solution with 0.2 M HCl or 0.5 M KNO_3_

Trp solution	*e*_(aq)_^–^	triplet	L_a_	cation	other
(aq)	719 (320)	430 (74)	450 (77)	580 (104)	510 (58)
0.2 M HCl	719 (320)	415 (74)	450 (77)	580 (104)	510 (58)
0.5 M KNO_3_	719 (320)	430 (74)	450 (77)	580 (104)	510 (58)

For 269 nm photoexcitation, globally fitting the kinetic traces of *e* ^–^(aq) and ^3^Trp to a biexponential decay function gave time scales of 3.0 ± 0.6 and 0.4 ± 0.2 ns that were then used as fixed parameters in fits to the Trp^+^, L_a_ and ‘other’ kinetic traces (Figure 5). Similar time scales were also obtained for fits to kinetic traces from TA spectra recorded following photoexcitation at both 289 and 263 nm (Figures S9 and S10). Thus, the time scales associated with the population of ^3^Trp are the same as those associated with the decay of the solvated electron, and those associated with Trp fluorescence in aqueous solution.^78^ The mean relative weightings of the longer time constants associated with the decay of the solvated electron and population of ^3^Trp obtained from our measurements at 289 nm, 269 and 263 nm, are 0.8 ± 0.1 and 0.7 ± 0.1 respectively, which are consistent with the relative weighting of the longer time constant associated with the fluorescence lifetime of aqueous Trp following 264 nm photoexcitation reported by Fleming and co-workers (0.78).^49^ The amplitudes of the shorter time constant decays obtained from our fits to the Trp^+^, L_a_ and ‘other’ kinetic traces, were zero, or significantly less than the amplitude of the longer component, for all photoexcitation wavelengths, which we attribute to the fact that the TA spectra are dominated by the contributions from the solvated electron and ^3^Trp.

Since the time scales and relative weightings associated with the decay of the solvated electron match those of the fluorescent L_a_ state, it seems that there must be a dynamic equilibrium between the L_a_ state and the cation-electron ion pair, L_a_ ⇌ Trp_(aq)_^+^ + *e*_(aq)_^–^. To explore this further, we investigated the effect on the fluorescence quantum yield of adding solvated electron quenchers, to see whether they drained the fluorescent L_a_ state. Fluorescence spectra recorded following 269 nm photoexcitation in aqueous solution, and in aqueous solution with 0.2 M HCl or 0.5 M KNO_3_ added, are presented in Figure 6. The fluorescence profiles do not change, but quenching the solvated electrons reduces the fluorescence yield considerably; the relative integrated areas of the fluorescence spectra in aqueous solution, with 0.2 M HCl added, and with 0.5 M KNO_3_, are 1, 0.18, and 0.03, respectively. This comparison supports the idea of a dynamic equilibrium between the fluorescent L_a_ state and the cation-electron ion pair.

**Figure 6:**
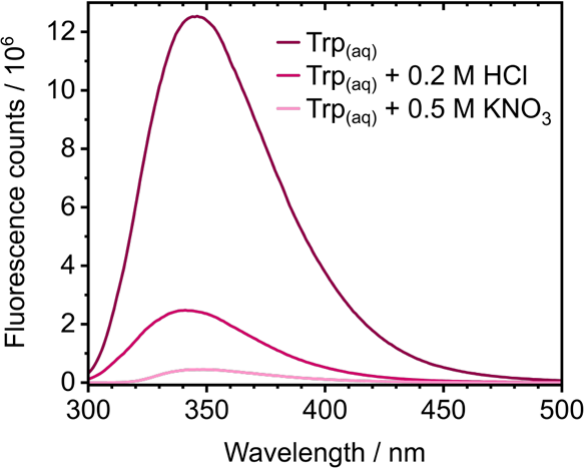
Fluorescence spectra of 0.5 mM Trp in aqueous solution and in aqueous solution with either 0.2 M HCl or 0.5 M KNO_3_ added, recorded following photoexcitation at 269 nm.

To explore the effect of adding solvated electron quenchers on the dynamics of triplet population, TA spectra of aqueous Trp with 0.2 M HCl or 0.5 M KNO_3_ added were recorded using the UCL laser facility. TA spectra recorded following photoexcitation at 269 nm are presented in Figure 7 and analogous spectra following photoexcitation at 289 nm are presented in the SI (Figure S11).

**Figure 7:**
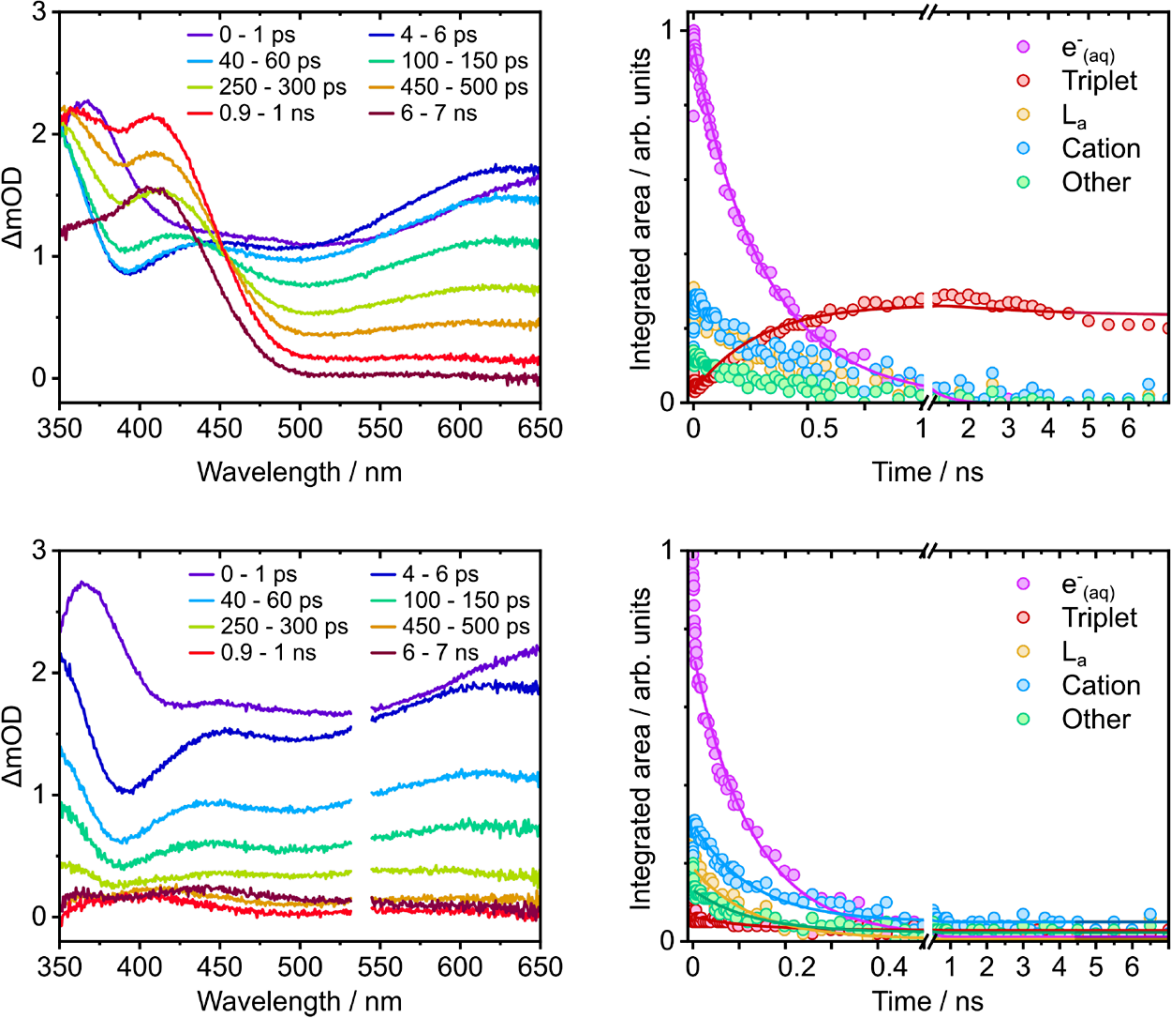
Left: Transient absorption spectra of 3.3 mM tryptophan at specified pump–probe delays following 269 nm photoexcitation in aqueous solution with the addition of 0.2 M HCl (top) or 0.5 M KNO_3_ (bottom, note that scattered light observed at twice the pump wavelength has been removed). Right: corresponding kinetic traces of the features in the transient absorption spectra identified in Table 2, with global biexponential fits to *e* ^–^(aq) and ^3^Trp TA features with one time constant fixed to 3 ns (top) and exponential fits to all features in the TA spectra (bottom).

For measurements with 0.2 M HCl added as a solvated electron quencher, the overall appearance of the spectra is similar to those in aqueous solution (Figure 3); however, the time scales are different. The bands associated with solvated electron absorption and the L_a_ ESA, decay more rapidly in solution with 0.2 M HCl added and the ^3^Trp ESA rises more rapidly. The ^3^Trp ESA is shifted to shorter wavelengths in spectra recorded with 0.2 M HCl added (415 nm) compared with aqueous solution (430 nm), consistent with earlier observations.^44^ By 7 ns, the solvated electron band has disappeared and the TA spectrum is dominated by the ^3^Trp ESA around 415 nm, an ESA around 365 nm which evolves on similar time scales and is therefore also assigned as a ^3^Trp ESA, and a minor contribution from the Trp^+^ absorption around 580 nm, in agreement with earlier nanosecond and femtosecond TA measurements.^45,67^ In contrast, for measurements with 0.5 M KNO_3_ added, the TA spectra have quite a different appearance. The solvated electron is quenched considerably more rapidly in 0.5 M KNO_3_ than 0.2 M HCl. The absorbance in the 400–500 nm range still appears to shift to shorter wavelengths, suggesting that some ^3^Trp is formed, although it is largely quenched, which is consistent with a formation mechanism involving the solvated electron.

To quantify the dynamics of triplet population in these quenching experiments, the 0–7 ns TA spectra were decomposed into the same five spectral bands that were fit to the aqueous solution TA spectra, over the range 430–660 nm. The only difference is that the triplet ESA band is shifted 15 nm to shorter wavelengths for the spectra recorded with 0.2 M HCl. The integrated areas of the five spectral bands following photoexcitation at 269 nm are plotted as a function of time in Figure 7. Analogous kinetic traces obtained from the 289 nm TA spectra are plotted in Figure S11.

For measurements with 0.2 M HCl added as a solvated electron quencher, global fits of the decay of the solvated electron absorption and rise of the ^3^Trp ESA to an exponential function gave a time constant of 308 ± 9 ps. This time constant yields a quenching rate constant of 1.6 × 10^10^ M^–1^ s^–1^, which is in agreement with the literature bimolecular scavenging rate of solvated electrons (1.5 × 10^10^ M^–1^ s^–1^ for 0.1 M H^+^).^89^ The maximum population of ^3^Trp is lower than in aqueous solution without HCl (Figure 5), and after around 2 ns when there is a negligible concentration of *e* ^–^(aq) remaining, the ^3^Trp ESA begins to decrease in intensity. Global fitting to a biexponential function gave a similar time constant of 308 ± 8 ps and a longer time constant of 4 ± 2 ns. There are contributions of the longer time scale in all the spectral features, and since the 3 ns fluorescence lifetime lies within the error bars of these longer time scale, we also tried fitting a biexponential function with one time constant fixed at 3 ns to both sets of HCl quenched data (Figures 7 and S11). The smaller time constant obtained from the constrained fit is similar to those obtained using the other methods (337 ± 9 ps).

For the measurements with 0.5 M KNO_3_ added as a solvated electron quencher, a fit of the decay of the solvated electron absorption gave a time constant of 133 ± 4 ps This time constant yields a quenching rate constant of 1.5 × 10^10^ M^–1^ s^–1^, which is close to a literature rate constant for solvated electron scavenging by NO_3_^–^ (9.7 × 10^9^ M^–1^ s^–1^).^90^ A global fit of all the features in the spectra to an exponential function gave a similar time constant of 127 ± 3 ps.

The weighted mean of the two time constants associated with solvated electron decay for Trp in aqueous solution, and the time constants associated with solvated electron decay when 0.2 M HCl and 0.5 M KNO_3_ have been added, have approximate relative values of 1, 0.16, and 0.06, which are in good agreement with the relative integrated areas of the fluorescence spectra presented in Figure 6 (1, 0.18, and 0.03, respectively).

To quantify the dynamics of our triplet quenching measurements (Figure 4), the 290 nm TA spectra were decomposed into ^3^Trp, L_a_ and Trp^+^ bands (Table 2) over the range 430–495 nm for 0–8 ns, and fit with just the ^3^Trp band over the range 410–490 nm for longer times (12 ns–8.3 μs). In the fit to longer times, the center of the ^3^Trp band was allowed to float to fit the monotonic shift toward shorter wavelengths that is observed during the first 100 ns. Such a shift could arise from a slow decay of the underlying solvated electron absorption band that was not included in this fit, or dynamics on the triplet potential energy surface. Snapshots of the KOALA fits are presented in Figures S12 and S13. The 0–8 ns kinetic traces fit well to time constants of 3 and 0.6 ns representing the fluorescence lifetimes (Figure S14). Fits of the 12 ns −8.3 μs kinetic traces associated with the decay of the ^3^Trp ESA to biexponential functions gave time constants of 37 ± 3 ns and 4 ± 2 μs in aqueous solution, and 38 ± 3 and 430 ± 70 ns in aqueous solution with 0.2 M MnSO_4_ added (Figure 8). The 4 μs time constant obtained from measurements in aqueous solution is 3 orders of magnitude smaller than the phosphorescence lifetime of aqueous Trp;^14^ noting that our measurements were undertaken using relatively high concentrations of Trp and the solutions were not deoxygenated, we attribute this to competing quenching processes. The 430 ± 70 ns time constant obtained from measurements in aqueous solution with 0.2 M MnSO_4_ yields a rate constant of approximately 1 × 10^7^ M^–1^ s^–1^, which is close to a value reported for triplet quenching of 10^–4^ M melatonin by MnSO_4_ (5 × 10^7^ M^–1^ s^–1^).^91^ It is interesting that we also observe a ∼ 40 ns time constant, similar to earlier work by Bent and Hayon,^46^ and Haacke and co-workers.^45^ We attribute this to excited state dynamics such as a proton transfer, possibly for just one conformer, followed by intersystem crossing or quenching to repopulate the electronic ground state.

**Figure 8:**
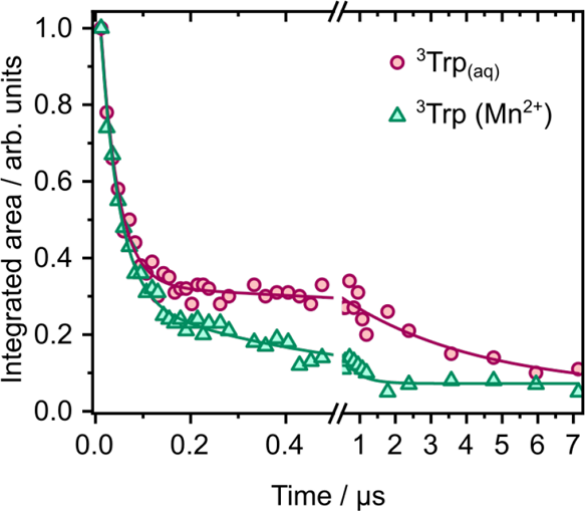
Kinetic traces of the integrated areas of the ^3^Trp ESA in the 290 nm TA spectra of 3.3 mM aqueous Trp and 3.3 mM aqueous Trp with 0.2 M MnSO_4_ added, with biexponential fits.

### Time-Resolved Infrared Spectroscopy

To complement our TA studies, we also recorded time-resolved infrared (TRIR) spectra of 3.3 mM Trp in D_2_O using the LIFEtime facility at RAL. TRIR spectra for 269 nm photoexcitation are presented in Figure 9 and those for 288 nm photoexcitation are presented in Figure S15. The positive feature centered at 1582 cm^–1^ that initially grows in before beginning to decay after around 10 ns is assigned to the ^3^Trp state. The negative feature centered at 1615 cm^–1^ matches the absorption in the FTIR spectrum attributed to the carbonyl stretch of the carboxylate group (Figure S16), and is a ground-state bleach (GSB). The intense positive feature centered at 1630 cm^–1^ that decays to around half its original intensity after 3 ns is assigned to the L_a_ state on the basis of quenching measurements that are discussed below.

**Figure 9:**
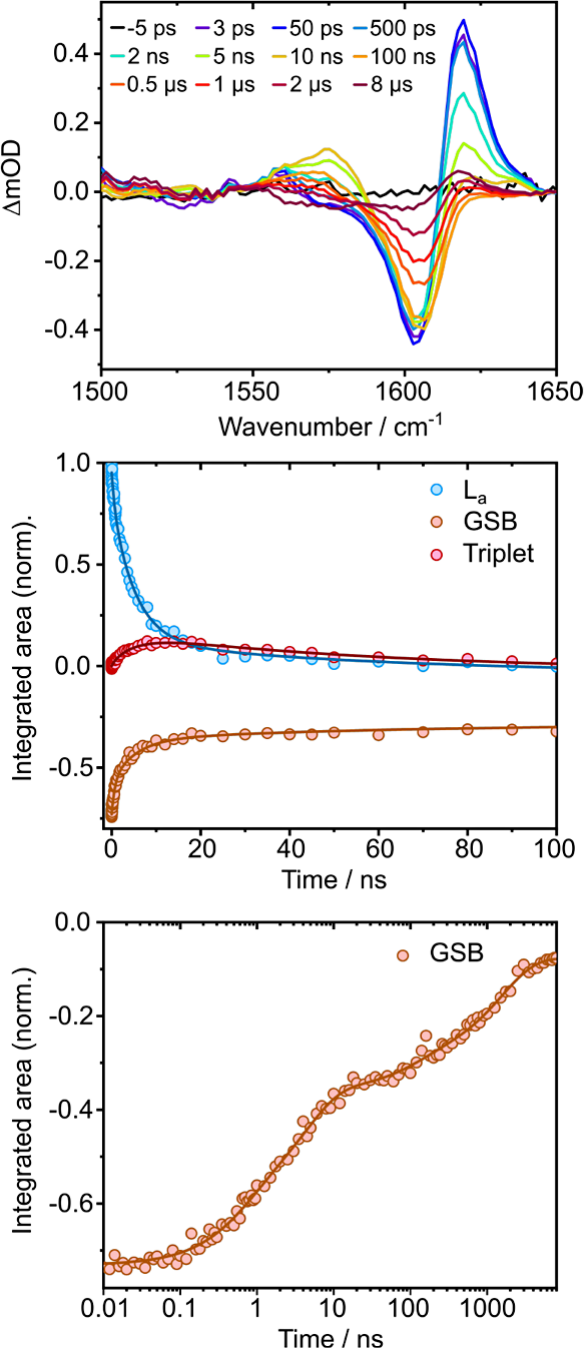
TRIR spectra of 3.3 mM tryptophan in D_2_O following photoexcitation at 269 nm at specified pump–probe delays from 3 ps to 8 μs (top). Corresponding kinetic traces of the integrated areas of features assigned to L_a_ (1630 cm^–1^), ^3^Trp (1582 cm^–1^) and a GSB (1615 cm^–1^) are globally fit to three exponentials over the range 0–100 ns (middle). The GSB is also fit to four exponentials over the range 0–8 μs (bottom).

The kinetic traces corresponding to the integrated areas of the L_a_ state, ^3^Trp and GSB features, determined by spectral decomposition using the KOALA software,^88^ were globally fit to three exponentials over the range 0–100 ns to give time scales of 0.7 ± 0.1, 5.2 ± 0.3, and 60 ± 12 ns for 269 nm photoexcitation (Figure 9). Similar time scales were obtained for global fits to kinetic traces obtained from the 288 nm TRIR spectra (Figure S15). The small 50–60 ns contribution to the decay of the vibrational feature assigned to the L_a_ state is attributed to contamination from the overlapping broad GSB feature during the spectral decomposition. These fits were made over the range 0–100 ns because the triplet ESA absorbance becomes negative at longer times. The TRIR bands sit on a broad, but weak, absorption band of D_2_O, and it is possible that as the photoexcited Trp molecules cool, the D_2_O heats and the absorption band shifts, leading to a negative baseline effect. Compared to our TA measurements, deuteration appears to have relatively little impact on the subnanosecond time scale, but increases the 3 ns time scale seen for experiments in H_2_O by a factor of ∼1.8 (1.7 and 1.9 for 269 and 288 nm photoexcitation, respectively). This observation is in agreement with deuterium-isotope fluorescence quantum yield enhancement factors reported in the literature, which lie in the range 1.65–2.3 at pH 7,^51,92–94^ and is consistent with the longer fluorescence lifetime being the major contribution (∼80%). The 50–70 ns time constants are consistent with the ∼ 40 ns time constants obtained from our 0–8.3 μs TA measurements in H_2_O (Figure 8). To investigate the effect of quenching the solvated electron, we compared TRIR spectra of Trp in D_2_O, and Trp in D_2_O with 0.2 M DCl added, recorded over the range 0–4 ns using the femtosecond laser facility in Bristol (Figure S17). The kinetic traces corresponding to the integrated areas of L_a_ state, ^3^Trp and GSB features in the spectra in D_2_O (1630, 1580, and 1616 cm^–1^) can be fit to the 0.7 ± 0.1 and 5.2 ± 0.3 ns time constants obtained from the longer time scale measurements made using the LIFEtime facility at RAL. The kinetic traces corresponding to the integrated areas of L_a_ state and GSB features in the spectra with 0.2 M DCl added (1745 and 1725 cm^–1^) were globally fit to an exponential with time scale 400 ± 30 ps, which is a factor of 1.3 longer than that observed in our TA measurements with HCl (Figure 7), most likely a result of deuterium-isotope substitution. It is this quenching measurement that allows us to assign the features at 1630 cm^–1^ (in D_2_O) and 1745 cm^–1^ (with 0.2 M DCl) as the L_a_ state, because Trp^+^ is not expected to be quenched.

Time constants determined by fitting four exponentials to the 269 nm GSB integrated areas over the range 0–8 μs are 0.6 ± 0.1 ns, 4.3 ± 0.7 ns, 100 ± 44 ns and 1.6 ± 0.2 μs (Figure 9). The first two time constants are in good agreement with those obtained from the global fits described above and can be attributed to ground-state recovery by fluorescence from the L_a_ state. The last two time constants are consistent with those obtained from our 0–8.3 μs TA measurements in H_2_O and support our proposal that the ∼ 40 ns time constant is associated with excited state dynamics that return population to the electronic ground-state.

### Photochemical Mechanism

Figure 10 summarizes the photochemical pathways and time scales determined from all our TA and TRIR spectroscopy measurements of aqueous Trp following photoexcitation of the first absorption band at wavelengths in the range 289–263 nm, over time scales ranging from 100s of fs to 8 μs.

**Figure 10:**
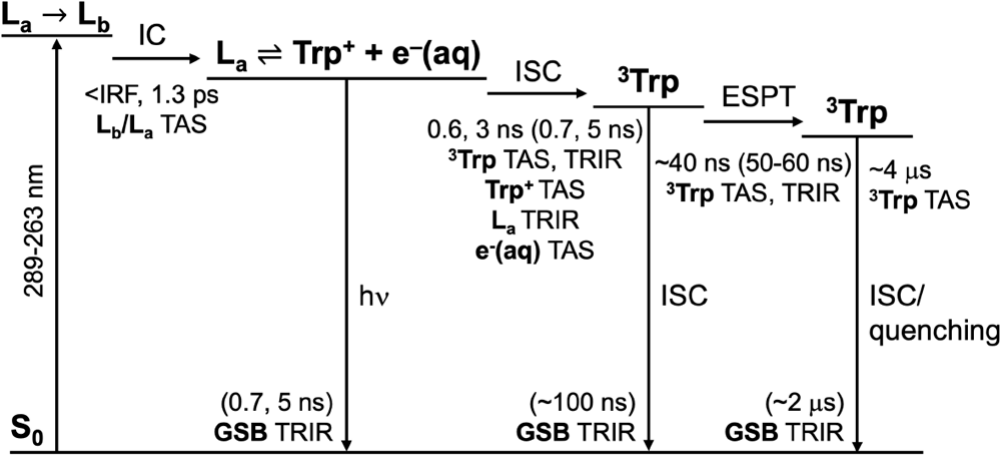
Photochemical pathway for relaxation of aqueous Trp following excitation at 289–263 nm. Experimental time constants are listed together with the spectral features from which they were obtained in TA and TRIR spectroscopy measurements; those in parentheses were determined from TRIR measurements in D_2_O rather than H_2_O.

Immediately following photoexcitation of the first absorption band of aqueous Trp, our TA spectroscopy measurements reveal rapid relaxation on time scales of <100 fs and ∼1.3 ps as well as evidence of a transfer of population from the L_b_ state to the L_a_ state, which is the adiabatic minimum in aqueous solution. These observations are consistent with ultrafast nonadiabatic population transfer from the initially populated L_a_ state to the lower lying L_b_ state followed by solvent-driven repopulation of the L_a_ state, as reported in a recent detailed study of the relaxation dynamics during the first few picoseconds following photoexcitation.^21^ We also observe prompt formation of electrons that become fully solvated within a few picoseconds. This is consistent with autoionization from the L_a_ or L_b_ state to form a cation-electron ion pair.

Subsequently, our TA spectroscopy measurements reveal a decay of cation and electron absorptions and the L_a_ ESA, with time scales that match the fluorescence lifetime of the L_a_ state of Trp in aqueous solution^49^ (∼3 and 0.6 ns in a ratio of approximately 4:1). This correspondence suggests that there is a dynamic equilibrium between the L_a_ state and the cation-electron ion pair, L_a_ ⇌ Trp^+^ + *e* ^–^(aq). Our measurements also reveal that population of ^3^Trp occurs on the same time scale as the decay of the cation and electron absorptions and fluorescence, indicating that ^3^Trp population is governed by the dynamic equilibrium between the L_a_ state and the cation-electron ion pair. Our TA measurements are supported by TRIR spectroscopy measurements of Trp in D_2_O that give time scales for ^3^Trp population, L_a_ state decay, and GSB recovery that are consistent with deuterium isotope fluorescence lifetime enhancement factors. Our longer time TA and TRIR spectroscopy measurements reveal that the ^3^Trp population relaxes with two time scales. The faster 40 ns time scale is consistent with earlier work,^45,46^ and we attribute this to excited state dynamics such as a proton transfer, possibly of just one conformer, followed by intersystem crossing back to the ground state. The remaining triplet state population relaxes on a longer time scale of a few μs, which is likely to be intersystem crossing or quenching.

Our proposal that ^3^Trp population is governed by the dynamic equilibrium between the L_a_ state and the cation-electron pair has some similarities with conclusions drawn from a recent detailed TA spectroscopy study of indole in aqueous solution.^72^ However, the triplet state of indole was observed to be populated on two time scales: ≤ 5 ns, similar to the 4.5 ns fluorescence lifetime of the L_a_ state; and a 2 orders of magnitude slower time scale of around 110 ns.^72^ This was explained in terms of a dynamic equilibrium between the L_a_ state of indole and a close contact cation-electron pair, L_a_ ⇌ [Indole^+^;*e* ^–^]­(aq), which subsequently dissociates to form a fully separated cation and electron that later recombine to form triplet indole after a spin-flip.

It is possible that ^3^Trp could be formed by recombination of a fully separated cation and electron after a spin-flip,

La⇌Trp++e−(aq)⇌Trp3

If this were the case, we need to consider why in indole there would be a dynamic equilibrium between a close contact cation-electron pair and the L_a_ state, whereas in Trp there would be a dynamic equilibrium between a fully separated cation-electron pair and the L_a_ state. The formation of solvated electrons is accompanied by a reorganization of solvent molecules, and the microenvironment of Trp, with its zwitterionic alanyl side chain, will be different to that of indole. Protein science studies have found rates of through-bond and through-space electron transfer, from the indole chromophore of Trp to the carbonyl in the peptide bond, to be closely linked to the conformation and environment of the Trp residue and its fluorescence lifetime.^95^ It is plausible that in aqueous solution, the rates of autoionization into bulk solvent and recombination could be enhanced by the alanyl side chain of Trp.

An alternative mechanism for forming ^3^Trp that is also compatible with the formation occurring on the same time scales as fluorescence and merits consideration is conventional intersystem crossing (ISC) from the L_a_ state,

Trp3←La⇌Trp++e−(aq)

If we assume that the two lowest lying triplet states have ^3^L_a_ and ^3^L_b_ character, El-Sayed’s rule would suggest that conventional ISC is inefficient. However, other factors such as the energy gap between the singlet and triplet states, the strength of spin–orbit coupling, and the molecular geometry are also important.^96–98^ It is conceivable that the electronic and structural changes resulting from the alanyl side chain could enhance the ISC rate.

Both mechanisms for ^3^Trp population are consistent with our TA and TRIR measurements. They are also both consistent with our electron quenching measurements in which the fluorescence quantum yield is reduced and, for HCl, the rate constant for triplet population matches the quenching rate constant.

## Conclusions

Transient absorption spectra measured over femtosecond to microsecond time scales for aqueous solutions of tryptophan photoexcited at UV wavelengths reveal bands characteristic of the Trp L_a_ state, the Trp^+^ radical cation, and solvated electrons. Spectral decomposition and kinetic fitting show that these bands decay on time scales consistent with the known fluorescence lifetimes of aqueous Trp. On matching time scales, our TA spectra also reveal growth of a band centered at 430 nm (blue-shifted to 415 nm for pH 1 solution) that we assign to the triplet state of Trp on the basis of quenching experiments. These observations reveal that Trp autoionization and electron-cation recombination play a key role in population of the Trp triplet state. Two mechanisms are proposed, which are indistinguishable in our TA and TRIR measurements. One is that, following autoionization, recombination of a radical cation and an electron with parallel spin forms the triplet state. In the other, recombination of the two species with opposing spins repopulates the Trp L_a_ state, from which the triplet state is populated by intersystem crossing. Regardless of whether one or other mechanism dominates, the measurements reported here show that an autoionization and electron-cation recombination mechanism, previously proposed for growth of triplet-state indole following UV excitation in aqueous solution, controls population of the triplet state in photoexcited aqueous Trp.

## Experimental Methods

L-tryptophan (Trp, ≥99% Sigma-Aldrich) was purchased and used without further purification. 3.3 mM solutions of aqueous Trp were made using deionized water. Hydrochloric acid (HCl, 37% Sigma-Aldrich), potassium nitrate (KNO_3_, ≥99%, Sigma-Aldrich) and manganese­(II) sulfate monohydrate (MnSO_4_ · H_2_O, ≥99%, Sigma-Aldrich) were purchased and used without further purification. 0.2 M solutions of HCl, KNO_3_ and MnSO_4_·H_2_O were made using deionized water. UV–visible absorption spectra were recorded using a Shimadzu UV-3600i Plus spectrophotometer and fluorescence spectra were recorded using Horiba Fluorolog-3 spectrofluorometer. NMR studies of 1, 3.3, and 40 mM aqueous Trp illustrate negligible aggregation in 3.3 mM solutions.

Femtosecond TAS experiments were carried out using the UCL Ultrafast Laser Facility, the Bristol femtosecond TRIR spectroscopy setup, and the LIFEtime Facility at the STFC Rutherford Appleton Laboratory. The UCL facility has been described previously.^86,99^ Briefly, femtosecond laser pulses were generated by a Ti:Sapphire oscillator regenerative amplifier system (Coherent Astrella-HE-USP). For UV excitation, tunable femtosecond laser pulses were generated by an optical parametric amplifier (Coherent OPerA Solo). Transient absorption spectra were recorded using a commercial transient absorption spectrometer (Ultrafast Systems HELIOS Fire) following photoexcitation at 289, 269, and 263 nm with pulse energies of 150 nJ at the sample. The focusing conditions and these pulse energies were set to avoid multiphoton ionization of solvent water. The broadband UV–visible probe (340–660 nm) was generated by focusing a small portion of the 800 nm fundamental from the Astrella into a calcium fluoride crystal. The relative polarizations of the pump and probe beams were set at the magic angle of 54.7°. The pump (modulated at 500 Hz) and probe were then focused into the center of a Harrick cell fitted with 2 mm CaF_2_ windows separated by a 250 μm PTFE spacer. A peristaltic pump (Masterflex 77912-10) continuously flowed sample solution at 32 mL min^–1^ (150 rpm) through the Harrick cell. After transmission through the sample, the probe signal was directed to an optical fiber-coupled detector. Transient spectra were processed using functions built into the Surface Xplorer software, including chirp correction and the subtraction of background noise.

TRIR measurements at the University of Bristol used an amplified ultrafast Ti:sapphire laser system (Coherent Astrella, 7W, 1 kHz, 35 fs pulse duration) to pump two OPAs (Coherent OPerA Solo). One OPA generated UV pump pulses, and the second produced broadband (300 cm^–1^) mid-IR probe pulses. The pump and probe pulses were spatially overlapped at a Harrick cell fitted with CaF_2_ windows separated by 100 μm. The samples were continuously circulated from a reservoir using a peristaltic pump. The linear polarizations of the pump and probe were set at magic angle (54.7°). Delays of up to 4 ns were controlled by a retro-reflecting mirror mounted on a movable stage (Thorlabs, DDS600/M) located in the UV pump beamline. After the sample, the mid-IR probe pulses were dispersed onto a liquid-N_2_ cooled 128-element MCT array detector (MCT-1-128, Infrared Associates Inc.) mounted in a spectrometer (Horiba Scientific, iHR320) and connected to fast readout electronics (Infrared Systems Development Corp., FPAS-0144). Reference IR pulses that bypassed the sample were collected in a matched spectrometer and detector, and used in data processing to improve signal-to-noise ratios. All the mid-IR beamlines were enclosed and purged with dry nitrogen. Further details of the experimental setup are described elsewhere.^100^

The LIFEtime Facility has been described previously.^101,102^ Briefly, UV pump pulses, and UV and visible-near-IR (for TAS) or mid-IR (for TRIR) probe pulses were generated by a synchronized pair of Yb-KGW amplifiers (Light Conversion, PHAROS 100 kHz, 15 W, 260 fs output pulses and PHAROS 100 kHz, 6 W, 180 fs output pulses) seeded by a common Yb:KGW ultrafast oscillator and pumping three OPAs (Light Conversion, ORPHEUS), one of which was used for UV excitation. The other two OPAs were used to generate separately wavelength tunable mid-IR pulses by difference frequency generation (DFG). For experiments at 269 nm, 288 and 290 nm excitation wavelengths, the pump pulse energies at the sample were 40, 150, and 17 nJ respectively. For TAS, a broadband probe in the visible-near-IR region (490–900 nm) was generated by focusing the 1030 nm output of the Yb laser into a sapphire crystal. A broadband probe in the UV region (350–420 nm) was generated by focusing the frequency-doubled output of the Yb laser (515 nm) into a CaF_2_ crystal. A 12 ns optical delay stage and further electronic delays out to μs time scales controlled the timing between the pump and probe pulses, prior to their spatial overlap at the center of a Harrick cell containing the sample. A peristaltic pump (Masterflex 77912–10) continuously circulated sample solutions through the Harrick cell, which was fitted with CaF_2_ windows separated by 250 μm spacers. The transmitted probe pulses were dispersed onto either a Si-array detector (Teledyne Octoplus) for TAS, or two 128-element MCT array detectors (Infrared Associates) for TRIR. Mid-IR pixel-to-wavenumber calibrations used reference spectra of polystyrene.

## Supplementary Material



## Notes

Transient absorption (TA) spectra; deconvolution of TA spectra and kinetic traces of the integrated areas of features in the spectra; time-resolved infrared spectra (TRIR); and FTIR spectra; kinetic traces of the integrated areas of features in the TRIR spectra (PDF)
