# Supplementary material for: Dynamic Equilibrium between the Fluorescent State of Tryptophan and Its Cation-Electron Ion Pair Governs Triplet State Population
Source: J Am Chem Soc. 2025 Aug 21;147(35):32064–76. doi: 10.1021/jacs.5c10445 (PMC12412166; doi:10.1021/jacs.5c10445)
Supplement: Supplementary file 1 [file ja5c10445_si_001.pdf]

Supporting Information: Dynamic Equilibrium Between the Fluorescent State of Tryptophan and its Cation-Electron Ion Pair Governs Triplet State Population

Rhea Kumar,[†,‡] Sufiyan Khan,[†,‡] Deborin Ghosh,[¶] Gabriel Karras,[§] Ian P. Clark,[§] Gregory M. Greetham,[§] Thomas A. A. Oliver,[¶] Andrew J. Orr-Ewing,[¶] and Helen H. Fielding[*,†]

†*Department of Chemistry, University College London, 20 Gordon Street, London WC1H 0AJ, United Kingdom*

‡*R. K. and S. K. contributed equally to this work*

¶*School of Chemistry, University of Bristol, Cantock's Close, Bristol BS8 1TS, United Kingdom*

§*Central Laser Facility, STFC Rutherford Appleton Laboratory, Didcot, Oxfordshire OX11 0QX, United Kingdom*

E-mail: h.h.fielding@ucl.ac.uk

Contents

S1 269 nm TA spectra supporting solvated electron assignment **S1**

S2 289 nm and 263 nm TA spectra of aqueous Trp **S2**

S3 Kinetic fits for 269 nm TA spectra: 0-18 ps **S4**

S4 Kinetic fits for 269, 289 and 263 nm TA spectra: 0-7 ns **S6**

S5 TA spectra and kinetic fits for 289 nm solvated electron quenching experiments **S12**

S6 Triplet quenching **S13**

S7 TRIR measurements **S16**

References **S19**

S1. 269 nm TA spectra supporting solvated electron assignment

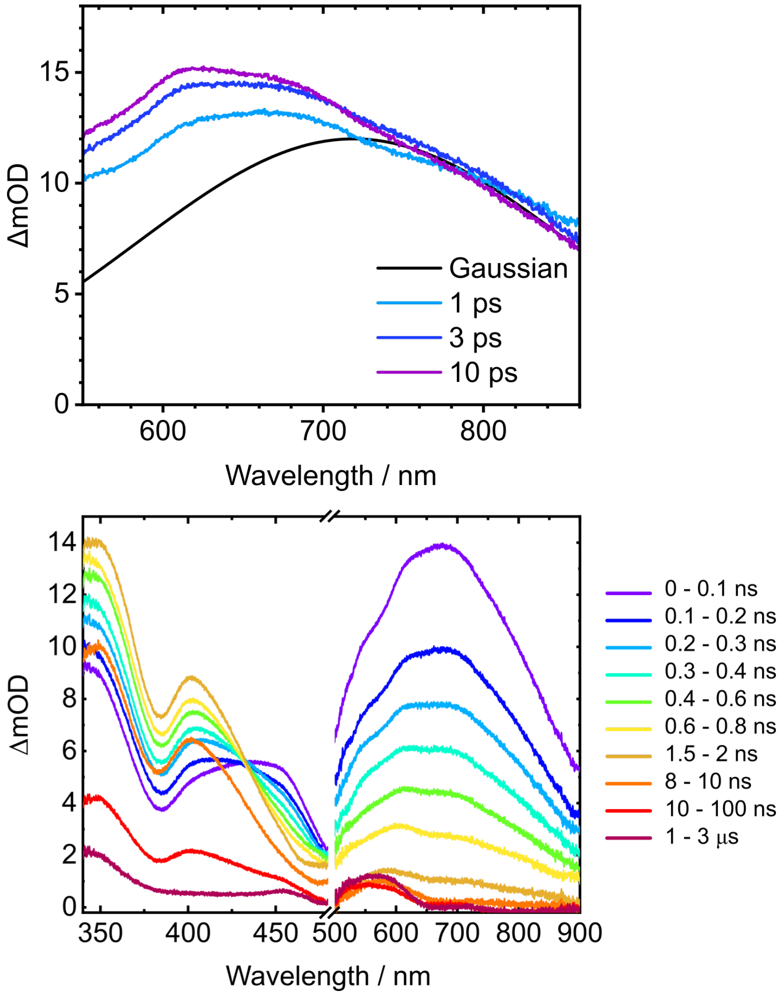

Figure S1: Top: transient absorption spectra of 3.3 mM aqueous Trp following 269 nm excitation at selected pump-probe delays plotted together with a Gaussian centered at 719 nm with FWHM 320 nm, to model the solvated electron absorption band.[S1,S2] Bottom: transient absorption spectra of 3.3 mM aqueous Trp following 269 nm excitation at selected pump-probe delays, with HCl added as a solvated electron quencher.

S2. 289 nm and 263 nm TA spectra of aqueous Trp

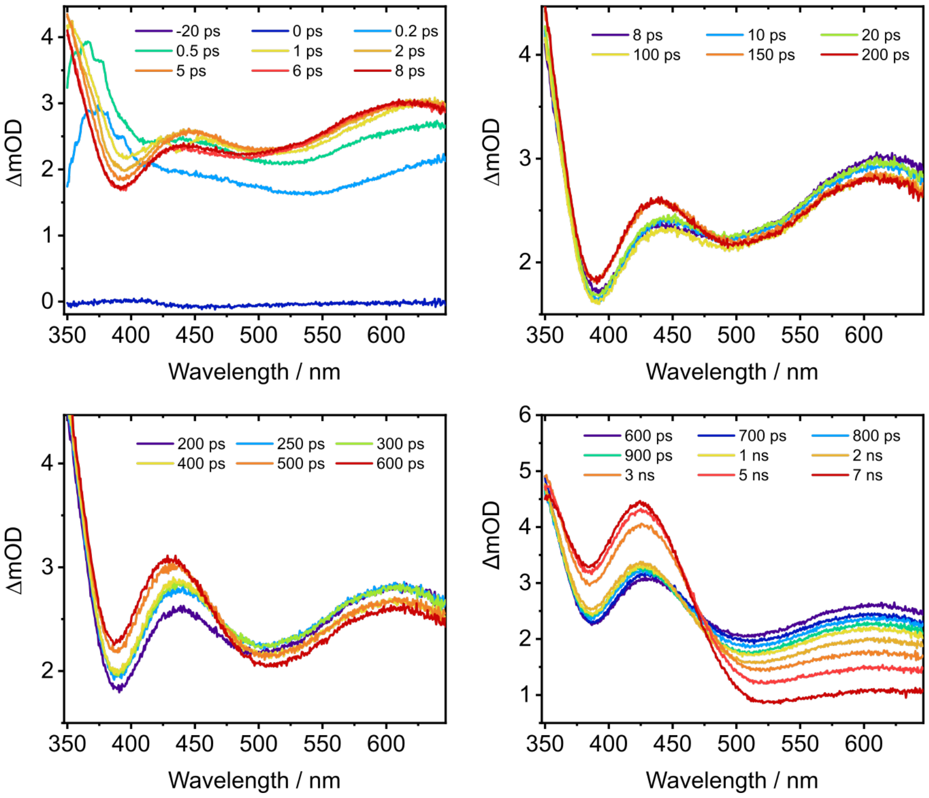

Figure S2: Transient absorption spectra of 3.3 mM aqueous tryptophan following photoexcitation at 289 nm at specified pump-probe delays, plotted over four ranges: 0-8 ps, 8-200 ps, 200-600 ps, and 0.6-7 ns.

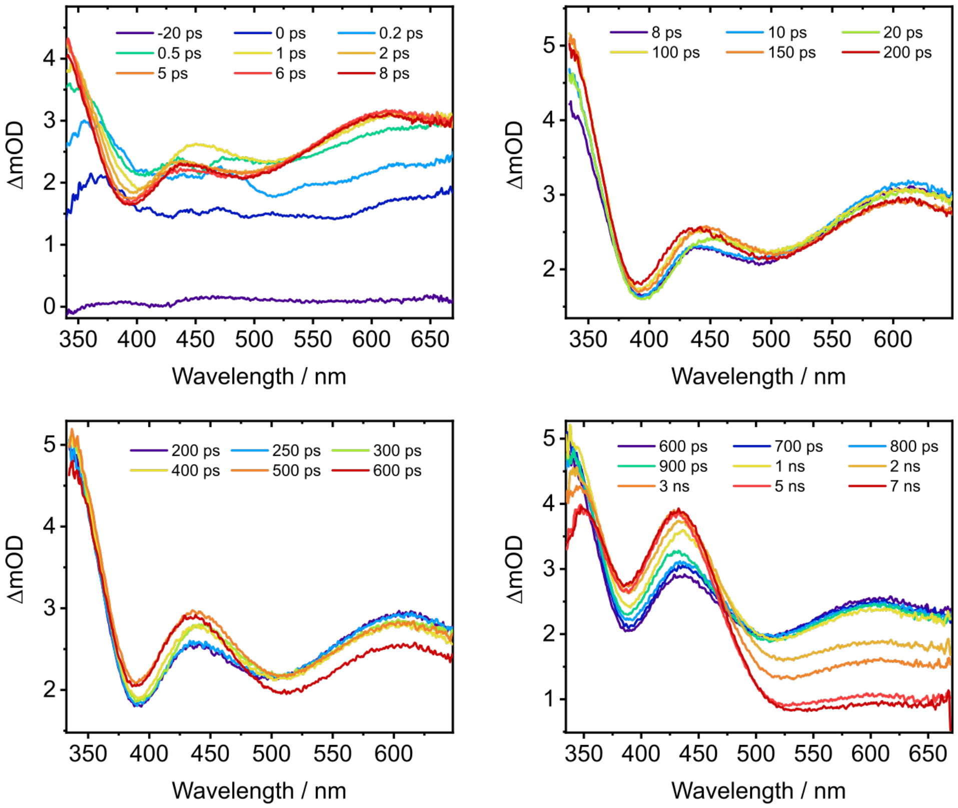

Figure S3: Transient absorption spectra of 3.3 mM aqueous tryptophan following photoexcitation at 263 nm at specified pump-probe delays, plotted over four ranges: 0-8 ps, 8-200 ps, 200-600 ps, and 0.6-7 ns.

S3. Kinetic fits for 269 nm TA spectra: 0-18 ps

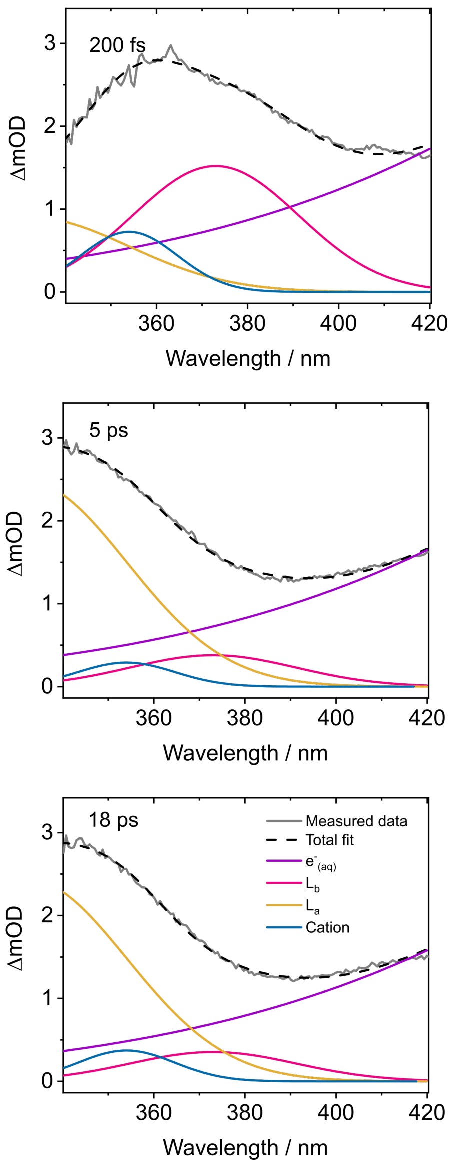

Figure S4: Deconvolution of transient spectra of 3.3 mM aqueous tryptophan following 269 nm excitation at specified pump-probe delays in the 0-18 ps range.

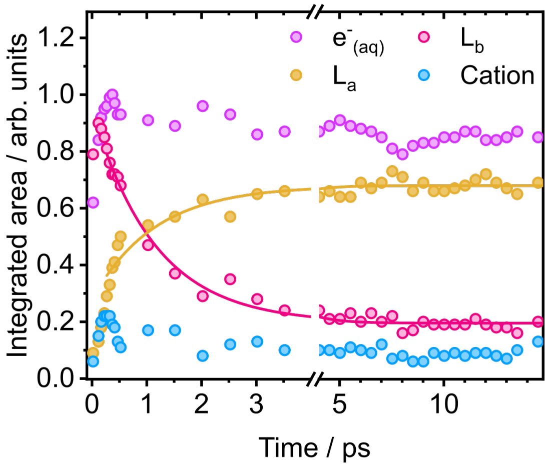

Figure S5: Kinetic traces of the features in the 269 nm TA spectra of aqueous Trp over the 0-15 ps range. The integrated areas of the L_b and L_a spectral bands have been globally fit to an exponential function for $t > 0.3$ ps, as described in the text.

S4. Kinetic fits for 269, 289 and 263 nm TA spectra: 0-7 ns

There are numerous overlapping spectral features in the TA spectra: solvated electron and Trp^+ absorptions, and ^3Trp, L_a and L_b ESAs. Moreover, the L_a and L_b ESAs are known to extend across the whole of the probe region of our UCL TA spectra.[S3] Nonetheless, over the range 430-660 nm and after the first few picoseconds when there is no longer any population remaining in L_b, the spectra could be decomposed into five spectral bands with Gaussian profiles whose central wavelengths and widths are presented in Table 2 of the paper. These Gaussians were selected to match the known solvated electron absorption maximum and width,[1] [S1,S2] and to represent a known Trp^+ absorption and known ^3Trp ESA (Results and Discussion), and a dominant L_a ESA.[S3] An additional absorption centered at 510 nm was included to improve the fit, and most likely accounts for an additional component of the L_a ESA. The Trp^+ absorption, ^3Trp ESA and L_a ESA parameters were optimised to fit the TA spectra of Trp in aqueous solution excited at all wavelengths reported in this work (289 nm, 269 nm and 263 nm), and also for Trp in aqueous solution with 0.2 M HCl, 0.5 M KNO_3, and 0.2 M $MnSO_4$, with the exception of the triplet ESA band which is shifted 15 nm to shorter wavelengths in TA spectra recorded with 0.2 M HCl, consistent with previous observations.[S4] Despite good fits to the TA spectra (Figures S6-S8), it should be noted that the Gaussians labelled as the solvated electron absorption and Trp^+ absorption bands likely include components of the L_a ESA. This does not affect the time constants determined from these fits because all these features decay with the same time constants over the 0-7 ns range. It may explain why the ratio of the Trp^+ absorption to solvated electron absorption observed in our deconvoluted spectra (Figs S6-S8) is different to that observed in similar TA measurements of indole,[S5] assuming that the Trp^+ and indole cation extinction coefficients

[1] This is an approximation because the absorption spectrum of the solvated electron is a Gaussian in energy and our fits employ Gaussians in wavelength.

are similar. Alternatively, this ratio could be different as a result of released electrons being quenched by recombination with the $-NH_3^+$ group of Trp^+.

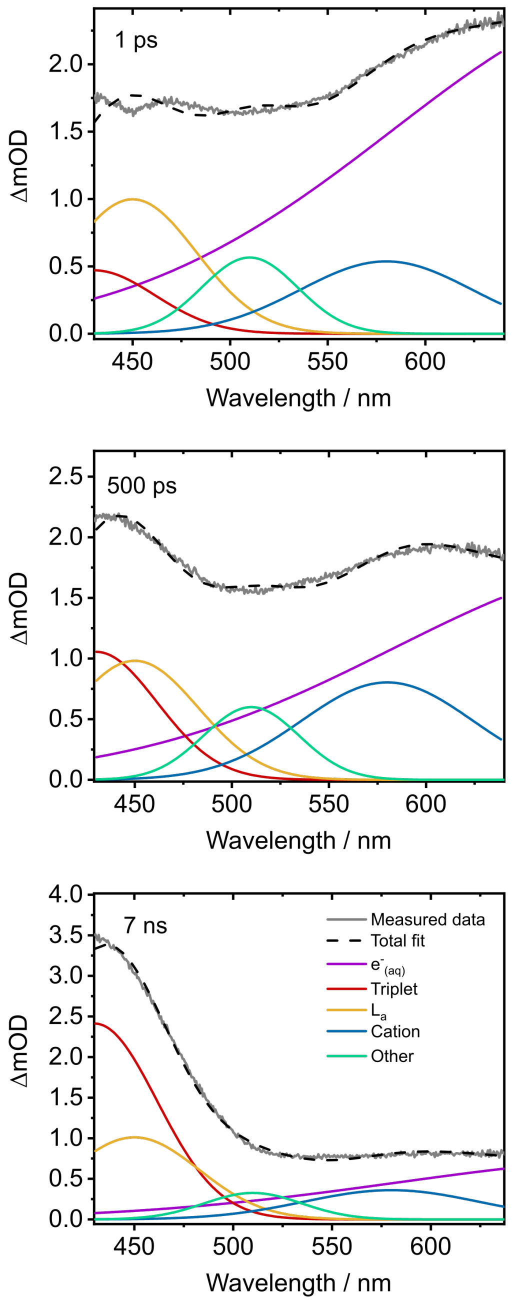

Figure S6: Deconvolution of transient spectra of 3.3 mM aqueous tryptophan following 269 nm excitation at specified pump-probe delays in the 0-7 ns range.

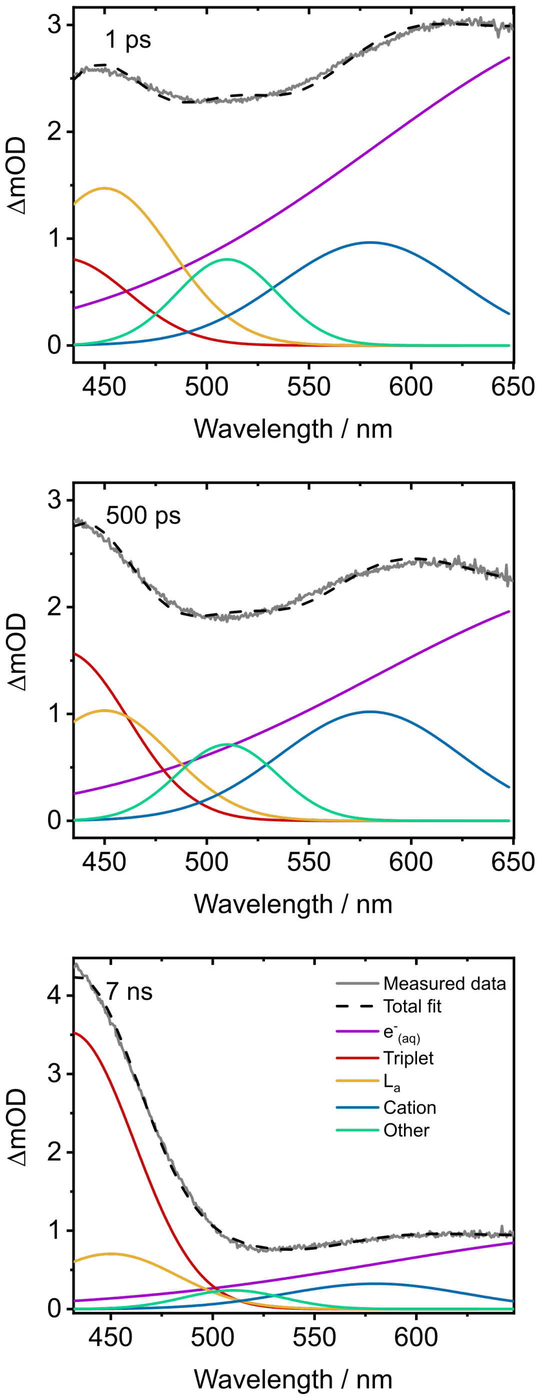

Figure S7: Deconvolution of transient spectra of 3.3 mM aqueous tryptophan following 289 nm excitation at specified pump-probe delays in the 0-7 ns range.

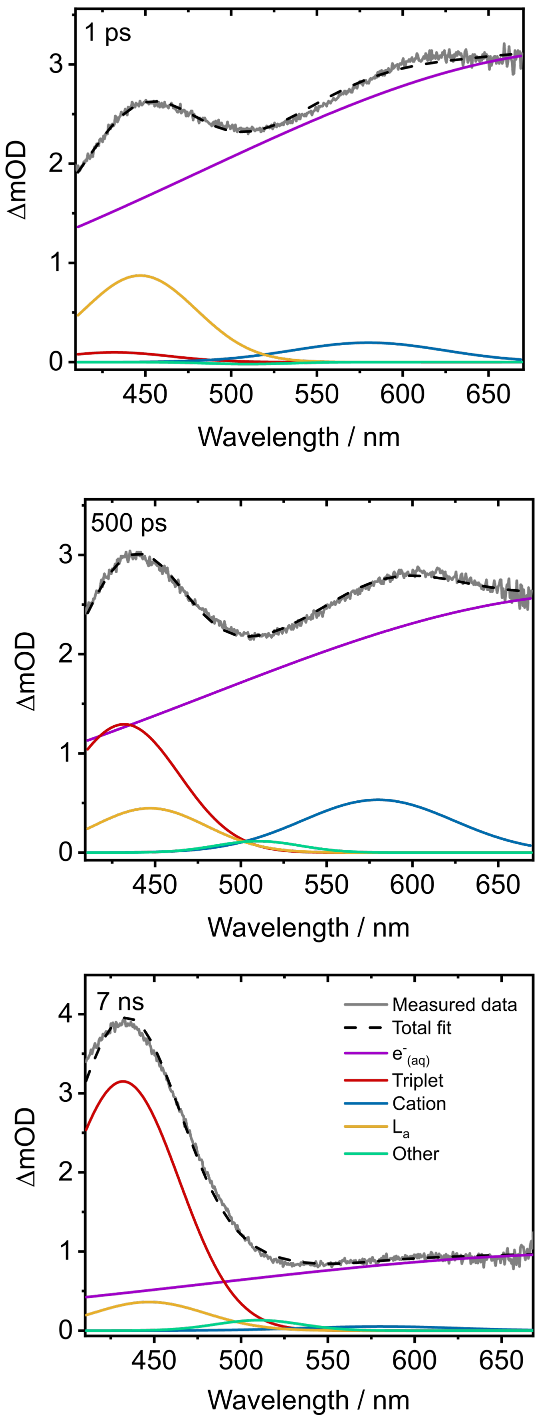

Figure S8: Deconvolution of transient spectra of 3.3 mM aqueous tryptophan following 263 nm excitation at specified pump-probe delays in the 0-7 ns range.

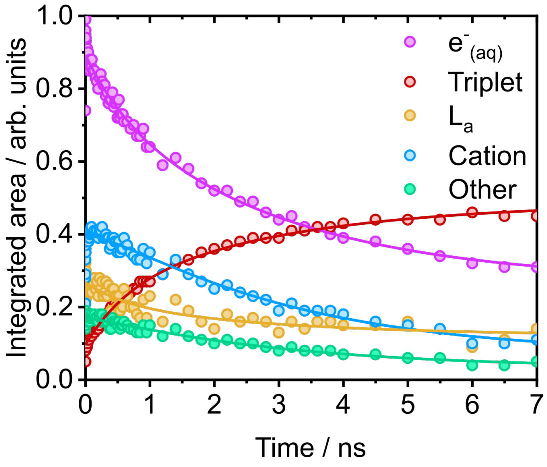

Figure S9: Kinetic traces of the features in the 289 nm TA spectra of aqueous Trp over the 0-7 ns range. The features of the TA spectra are fit with the parameters listed in the main text. The time-dependent integrated areas of the $e^-_{(aq)}$ and ^{3}Trp spectral bands are globally fit to a biexponential function giving time constants 3.1 ± 0.8 ns and 0.6 ± 0.2 ns. The integrated areas of the remaining three spectral bands are fitted to biexponential functions constrained to have the same time constants but amplitudes that were allowed to vary.

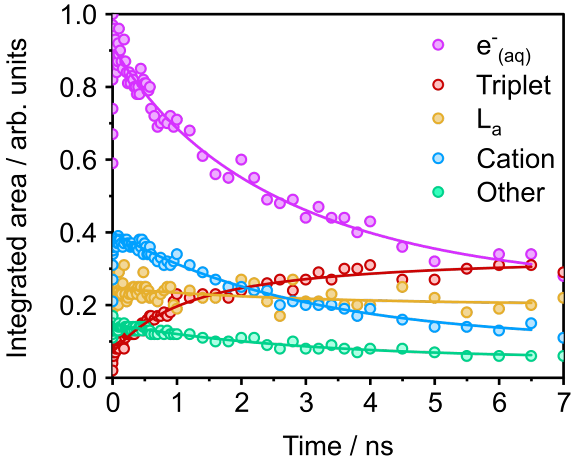

Figure S10: Kinetic traces of the features in the 263 nm TA spectra of aqueous Trp over the 0-7 ns range. The features of the TA spectra are fit with the parameters listed in the main text. The time-dependent integrated areas of the $e^-_{(aq)}$ and ^{3}Trp spectral bands are globally fit to a biexponential function, giving time constants 2.9 ± 0.8 ns and 0.8 ± 0.4 ns. The integrated areas of the remaining three spectral bands are fitted to biexponential functions constrained to have the same time constants but amplitudes that were allowed to vary.

S5. TA spectra and kinetic fits for 289 nm solvated electron quenching experiments

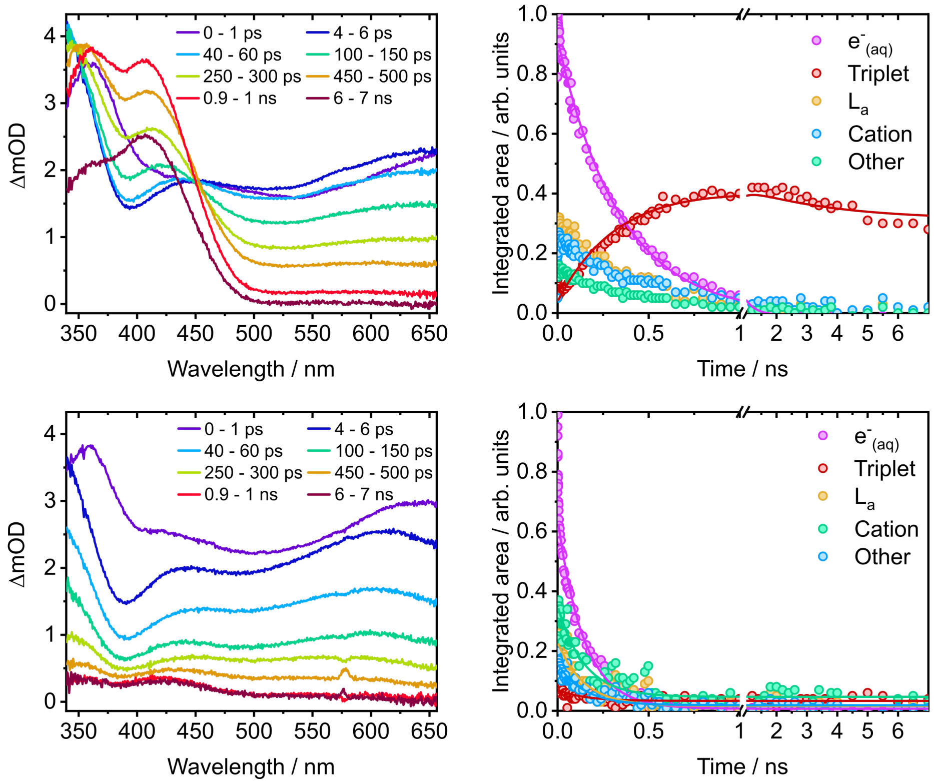

Figure S11: Left: transient absorption spectra of 3.3 mM tryptophan at specified pump-probe delays following 289 nm photoexcitation in aqueous solution with the addition of 0.2 M HCl (top) or 0.5 M KNO_3 (bottom, note that scattered light observed at twice the pump wavelength has been removed). Right: corresponding kinetic traces of the features in the transient absorption spectra with global bi-exponential fits to e^{-}(aq) and ^{3}Trp TA features with one time constant fixed to 3 ns, giving a second time constant of 299 $\pm$ 7 ps (top) and a global exponential fit to all features in the TA spectra giving a time constant 148 $\pm$ 5 ps (bottom).

S6. Triplet quenching

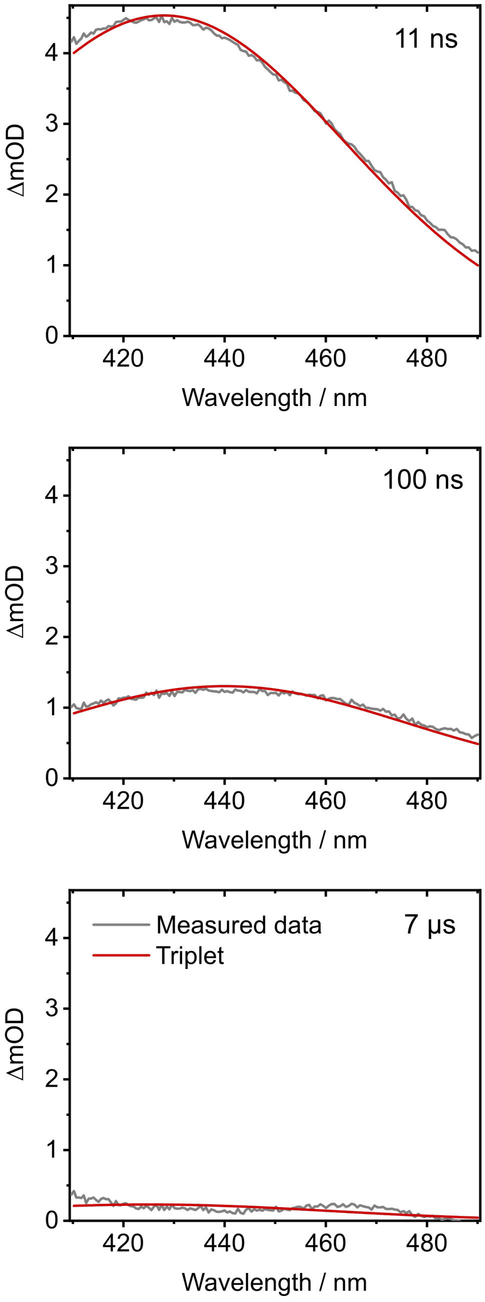

Figure S12: Deconvolution of transient spectra of 3.3 mM aqueous tryptophan following 290 nm excitation at specified pump-probe delays in the 0-7 μs range.

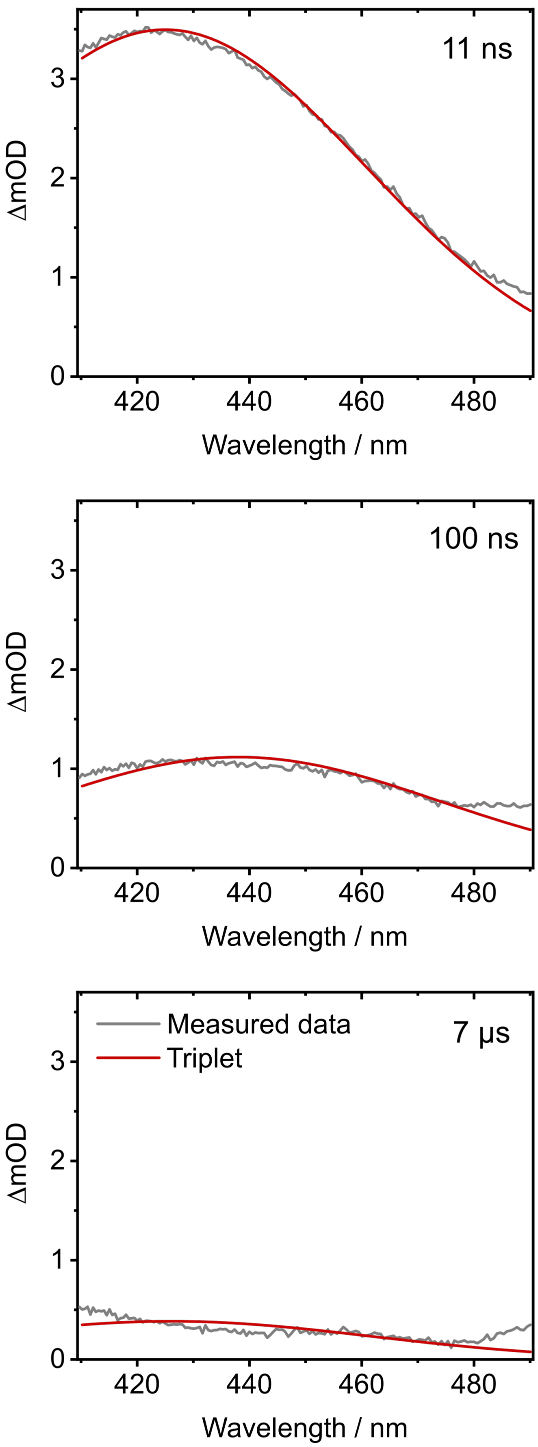

Figure S13: Deconvolution of transient spectra of 3.3 mM aqueous tryptophan with $MnSO_4$ following 290 nm excitation at specified pump-probe delays in the 0-7 μs range.

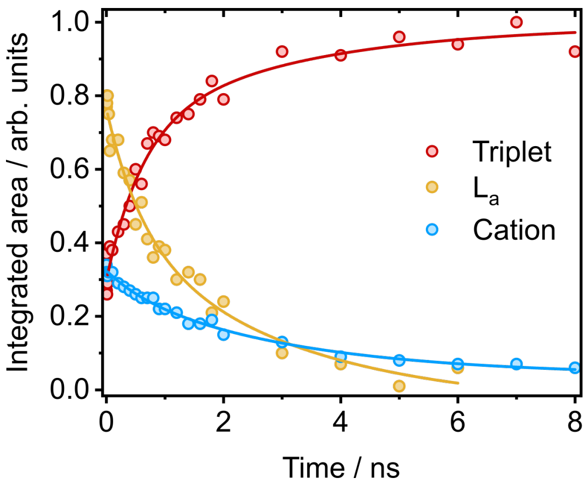

Figure S14: Kinetic traces of the features in the 290 nm TA spectra of aqueous Trp over the 0-8 ns range (bottom). The features of the TA spectra are fit with the parameters described in the main text. The time-dependent integrated areas of the ^{3}Trp, L_a and Trp^+ spectral bands are fit to time constants of 3 ns and 0.6 ns representing the fluorescence lifetimes.

S7. TRIR measurements

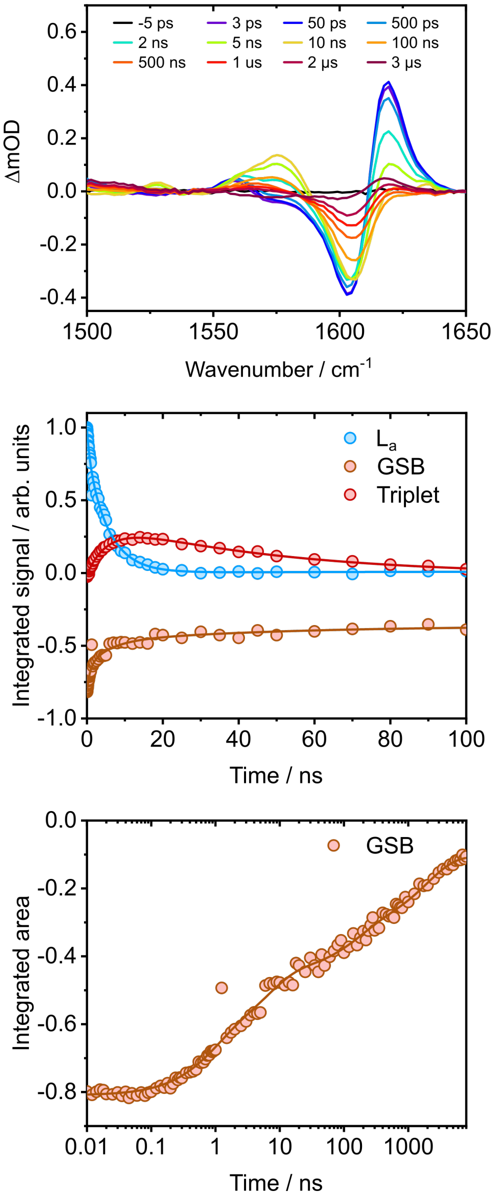

Figure S15: TRIR spectra of 3.3 mM tryptophan in D_2O following photoexcitation at 288 nm at specified pump-probe delays from 3 ps to 8 μs (top). Corresponding kinetic traces of the integrated areas of features assigned to L_a (1630 cm^{-1}), ^{3}Trp (1582 cm^{-1}) and a GSB (1615 cm^{-1}) are globally fit to three exponentials over the range 0-100 ns, giving timescales of 0.8 ± 0.1 ns, 5.6 ± 0.4 ns and 48 ± 13 ns (middle). The GSB is also fit to four exponentials over the range 0-8 μs (bottom).

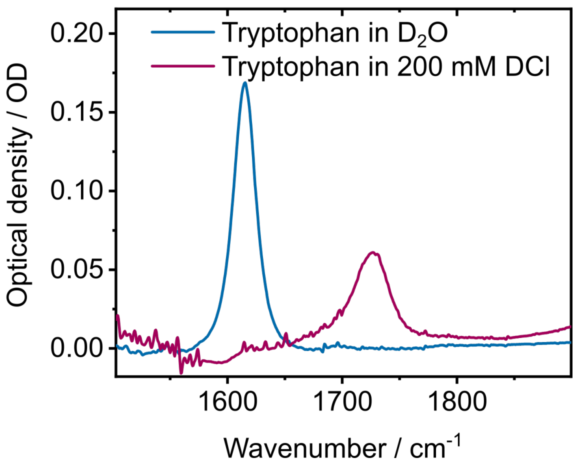

Figure S16: FTIR spectra of Trp in D_2O and in 200 mM DCl.

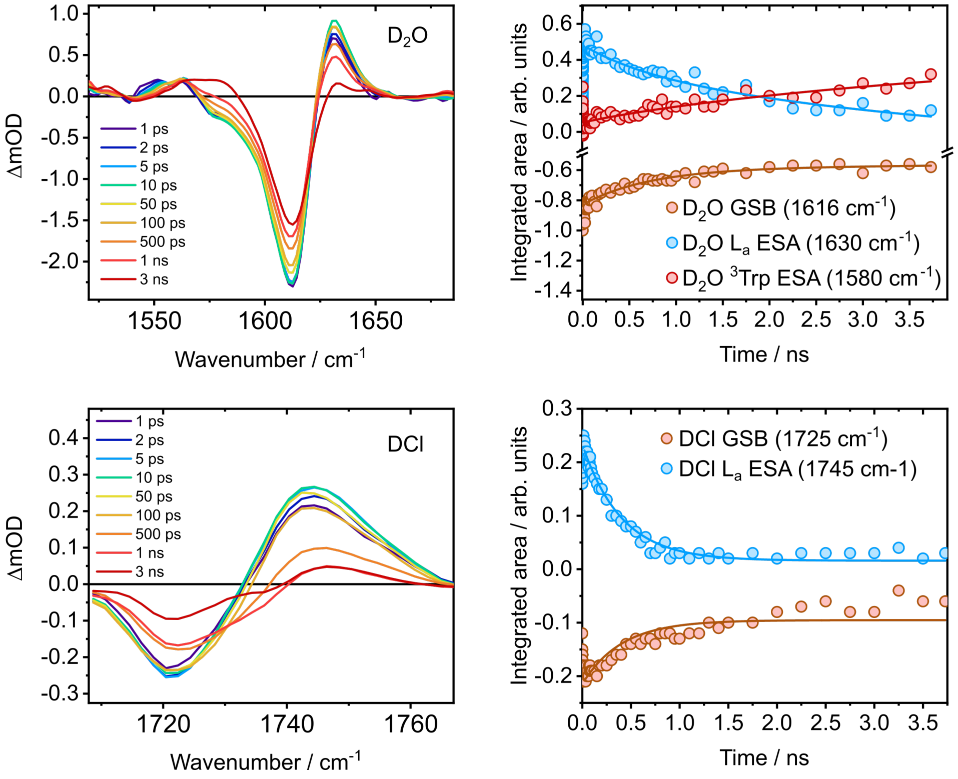

Figure S17: Left: TRIR spectra of Trp in D_2O (top) and 200 mM DCl (bottom), with corresponding kinetic traces of the integrated areas of features assigned to L_a, ^{3}Trp and a GSB. The kinetic traces corresponding to the integrated areas of the features in the spectra in D_2O are fit to 0.7±0.1 ns and 5.2±0.3 ns time constants. The kinetic traces corresponding to the integrated areas of Trp^+ and GSB features in the spectra with 0.2 M DCl added were globally fit to an exponential with time constant 400 ± 30 ps.

References

(S1) Baxendale, J. H.; Fielden, E. M.; Capellos, C.; Francis, J. M.; Davies, J. V.; Ebert, M.; Gilbert, C. W.; Keene, J. P.; Land, E. J.; Swallow, A. J.; Nosworthy, J. M. Pulse radiolysis. *Nature* **1964**, *201*, 468–470.

(S2) Oliver, T. A. A.; Zhang, Y.; Roy, A.; Ashfold, M. N. R.; Bradforth, S. E. Exploring Autoionization and Photoinduced Proton-Coupled Electron Transfer Pathways of Phenol in Aqueous Solution. *J. Phys. Chem. Lett.* **2015**, *6*, 4159–4164.

(S3) Jaiswal, V. K.; Kabacinski, P.; Nogueira de Faria, B. E.; Gentile, M.; de Paula, A. M.; Borrego-Varillas, R.; Nenov, A.; Conti, I.; Cerullo, G.; Garavelli, M. Environment-driven coherent population transfer governs the ultrafast photophysics of tryptophan. *J. Am. Chem. Soc.* **2022**, *144*, 12884–12892.

(S4) Sherin, P. S.; Snytnikova, O. A.; Tsentalovich, Y. P. Tryptophan photoionization from prefluorescent and fluorescent states. *Chem. Phys. Lett.* **2004**, *391*, 44–49.

(S5) Kumar, G.; Kellogg, M.; Dey, S.; Oliver, T. A. A.; Bradforth, S. E. Unraveling the photoionization dynamics of indole in aqueous and ethanol solutions. *J. Phys. Chem. B* **2024**, *128*, 4158–4170.
